# Boosting Immunity and Management against Wheat *Fusarium* Diseases by a Sustainable, Circular Nanostructured Delivery Platform

**DOI:** 10.3390/plants12061223

**Published:** 2023-03-08

**Authors:** Sara Francesconi, Riccardo Ronchetti, Emidio Camaioni, Stefano Giovagnoli, Francesco Sestili, Samuela Palombieri, Giorgio Mariano Balestra

**Affiliations:** 1 Department of Agriculture and Forest Sciences (DAFNE), University of Tuscia, Via San Camillo de Lellis, snc, 01100 Viterbo, Italy; 2 Department of Pharmaceutical Sciences, University of Perugia, Via del Liceo 1, 06123 Perugia, Italy

**Keywords:** fusarium head blight (FHB), fusarium crown rot (FCR), nanotechnology, cellulose nanocrystals (CNC), resistant starch, chitosan, gallic acid, plant resistance, plant elicitors, innate immunity

## Abstract

Fusarium head blight (FHB) and Fusarium crown rot (FCR) are managed by the application of imidazole fungicides, which will be strictly limited by 2030, as stated by the European Green Deal. Here, a novel and eco-sustainable nanostructured particle formulation (NPF) is presented by following the principles of the circular economy. Cellulose nanocrystals (CNC) and resistant starch were obtained from the bran of a high amylose (HA) bread wheat and employed as carrier and excipient, while chitosan and gallic acid were functionalized as antifungal and elicitor active principles. The NPF inhibited conidia germination and mycelium growth, and mechanically interacted with conidia. The NPF optimally reduced FHB and FCR symptoms in susceptible bread wheat genotypes while being biocompatible on plants. The expression level of 21 genes involved in the induction of innate immunity was investigated in Sumai3 (FHB resistant) Cadenza (susceptible) and Cadenza SBEIIa (a mutant characterized by high-amylose starch content) and most of them were up-regulated in Cadenza SBEIIa spikes treated with the NPF, indicating that this genotype may possess an interesting genomic background particularly responsive to elicitor-like molecules. Quantification of fungal biomass revealed that the NPF controlled FHB spread, while Cadenza SBEIIa was resistant to FCR fungal spread. The present research work highlights that the NPF is a powerful weapon for FHB sustainable management, while the genome of Cadenza SBEIIa should be investigated deeply as particularly responsive to elicitor-like molecules and resistant to FCR fungal spread.

## 1. Introduction

Bread wheat (*Triticum aestivum* L.) is one of the most cultivated cereals worldwide and the first source of protein for humans [1]. Biotic stresses are of particular concern for wheat production; in particular, *Fusarium* spp. are cosmopolitan, hemibiotrophic, fungal pathogens and infect bread wheat cultivars worldwide, since infections are favored by warm temperatures and high humidity conditions. Fusarium head blight (FHB) and Fusarium crown rot (FCR) are caused by a complex of *Fusarium* spp., where *F. graminearum* and *F. culmorum* or *F. pseudograminearum* are the predominant pathogens, respectively. FHB symptoms appear at the flowering stage as early bleaching of the spike. After direct and indirect penetration, the fungus spreads vascularly through the rachis, resulting in drastically decreased yield (up to 70%) and mycotoxin accumulation, such as deoxynivalenol (DON), nivalenol (NIV), and zearalenone (ZEN) into the kernels, which are chronically toxic for humans and animals [2]. FCR can occur from seed germination to milky ripening and can reduce grain yield by up to 35%. Typical symptoms are stem-based browning, leading to a reduced number of tillers, number and size of kernels, and premature death of the inflorescence [3]. Since *Fusarium* spp. are soil-borne pathogens and survive on crop residues, the infection occurrence is favored by minimum tillage; furthermore, the current climate change scenario is encouraging disease diffusion because of warmer temperatures [4]. FHB and FCR are mainly managed by applying synthetic fungicides, such as triazoles, benzimidazoles, or strobilurins. Triazoles and benzimidazoles can generally control for about 50–60% of *Fusarium* spp. diseases, while strobilurins are ineffective and can also favor the accumulation of mycotoxins in infected heads [5]. The main concerns regarding the application of synthetic fungicides are both agronomic and ecological: the current classes of fungicides do not translocate quickly, thus the application timing is crucial to be well individuated; moreover, farmers have difficulty with the timing of fungicide application, because favorable weather for FHB and FCR infection frequently coincides with conditions that are unfavorable for fungicide spraying [5]. Furthermore, the discovery of a tebuconazole-resistant strain of *F. graminearum* in the state of New York and of a benzimidazole-resistant strain in China poses a new challenge in the management of FHB and FCR by using conventional fungicides [6,7]. This discovery highlights the need to develop fungicides that are effective in reducing FHB and FCR, but with modes of action different from that of conventional ones. At the same time, synthetic fungicides affect the whole ecosystem; thus, the fauna and flora are continuously exposed to these substances, which are ingested and accumulated in the trophic chain, causing severe human and animal health problems [8]. In fact, severe restrictions have been posed on the use of compounds whose characteristics are persistence, bioaccumulation, toxicity, and long-range environmental transport [9]. As an example, as part of the Green Deal, the European Commission proposed to reduce the use and risk of chemical pesticides by 50% by 2030. To successfully achieve such an ambitious goal, an effective strategy to reduce conventional pesticides in agriculture is the employment of nanotechnology and natural active antimicrobials, which are substances of organic origin that limit the development of pathogens.

Nanotechnology has the concrete possibility of minimizing agrochemicals used in agriculture. Nanomaterials carriers can provide better coverage when applied on crops, enhancing antimicrobial properties thanks to a controlled release of bioactive compounds, and reducing the chemical dispersion in soil and waters. In this sense, a very effective low-cost solution is represented by organic polymers [10]. Among these, cellulose nanocrystals (CNC) are some of the most promising, due to their well-known biochemical properties, including high elasticity modulus, thermal stability, no cytotoxic effects, and high presence of hydroxyls groups, which can be exploited for the linking of active principles [11]. To grant controlled release features, starch can be also considered as an additive. Starch and different starch-based polymers are well-known excipients, frequently used in the food and pharmaceutical industries. Starch can facilitate the hydro-solubility of molecules by adsorbing water [12]. This ability is influenced by the content of amylose and amylopectin, which are normally present in a 1:3 ratio. Starch with high amylose (HA) content has a better film-forming ability and higher gelling strength compared to standard starch. Depending on origin and structure, HA starch shows useful properties in terms of modulation of active substance release [13]. Moreover, with the aim of improving human nutrition, research is currently focused on obtaining high amylose-content wheat genotypes. In particular, HA flour is positively correlated with resistant starch content that has been found to have many beneficial effects on human health, playing a similar role to dietary fiber [14]. In this scenario, CNC as nanocarriers and HA starch as a release modifier can be extremely useful to fabricate novel delivery systems linked to active antimicrobials able to boost growth, development, and innate defenses in wheat as well as to directly control FHB and FCR. In particular, antimicrobials and elicitor molecules are natural-based compounds able to show both direct and indirect inhibition effects on plant pathogens, by demonstrating toxicity against pathogens’ development while boosting systemic acquired resistance in plants [15]. Among this class of compounds, chitosan (CH) and gallic acid (GA) are organic-derived molecules extracted from the shells of shrimps and different plant tissues, respectively, and well-known for their biostimulant, antimicrobial, and elicitor-like properties [16,17]. In the present study, we investigated the potentialities of a novel nanostructured particle formulation (NPF), based on CNC and HA starch (HAS) obtained from wheat bran and containing CH and GA as active compounds, in improving FHB and FCR management and to boost innate immunity in wheat.

## 2. Results

### 2.1. CNC Synthesis, HAS Extraction, and Characterization from Cadenza SBEIIa Wheat Bran

CNC were successfully synthesized from cellulose extracted from the wheat bread genotype Cadenza SBEIIa. Table S1 shows that the average dry content (%) and yield (%) from the three extraction replicates were of 0.5% ± 0.3 and 15.3% ± 9.8. 

**Figure 1:**
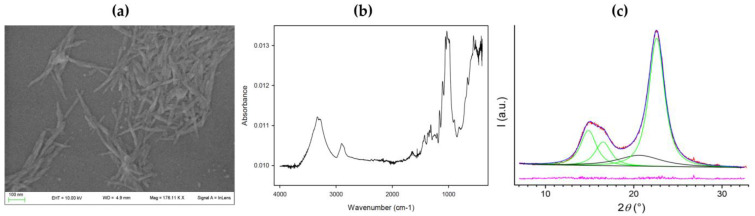
Characterization of CNC from wheat bran: (**a**) SEM picture showing typical acicular morphology; (**b**) ATR-IR profile with classical CNC signals; (**c**) XRD profile where the contributions of Iβ cellulose (green) and the amorphous (black) phases are highlighted. The experimental profile is in red, the calculated one is reported in blue, while their difference is shown at the bottom (pink). Total area = 1,208,154; Amorphous area = 159,617% crystallinity (cellulose Iβ) = 87.

SEM images evidenced typical acicular needle-shaped structures of about 200 nm (Figure 1a). The TR-IR profile confirmed classical CNC structure with no foreign signals (Figure 1b). The large band of CNC at 3600–300 cm^−1^ corresponds to the O-H stretchings, including intermolecular hydrogen bonds, while the band around 2900 cm^−1^ to the C-H and CH_2_ stretchings. In the spectrum, the peaks at 1428, 1316, 1034, and 898 cm^−1^, assigned to CH_2_ bending, CH_2_ rocking, C-O stretching, and C-H or CH_2_ bending, respectively, represent typical cellulose absorption peaks. On the other hand, XRD profiles of CNC extracted from wheat bran revealed a high crystallinity index of 87%. The crystal structure was characterized by the contribution of the only Iβ cellulose form (Figure 1c).

High-amylose starch (HAS) was already extracted and characterized in previous research works [14,18].

### 2.2. Synthesis and Characterization of the Nanostructured Particle Formulation (NPF)

The CNC from wheat bran were employed to synthesize aminated CNC (CNC-NH_2_). The obtained CNC-NH_2_ were evaluated for their capacity to bind GA and grant effective GA loading in the final formulation. GA loading efficiency on CNC-NH_2_ ranged between 50% and 70% (Table S2). GA content increased nearly proportionally with the CNC-NH_2_ amount added, confirming the effective enhancement of GA binding to CNC and an increase in the amount of active compound loaded in the final formulation.

The obtained CNC-NH_2_/GA was then combined with CH, Tween 80, and high-amylose starch (HAS) in order to obtain the desired final NPF. The yield was from 42% to 63% (Table S3). The average content of GA increased with the batch size processed and reached nearly 3% *w*/*w*, while CH remained nearly constant with an average of 25% *w*/*w*. Recovery was generally low with a clear effect of the batch size processed that led to nearly 50% for GA and 70% for CH. This parameter was affected by the physiological yield of the spray-drying process that, however, was comparable to the average performances recorded with this technique even in other fields.

**Figure 2:**
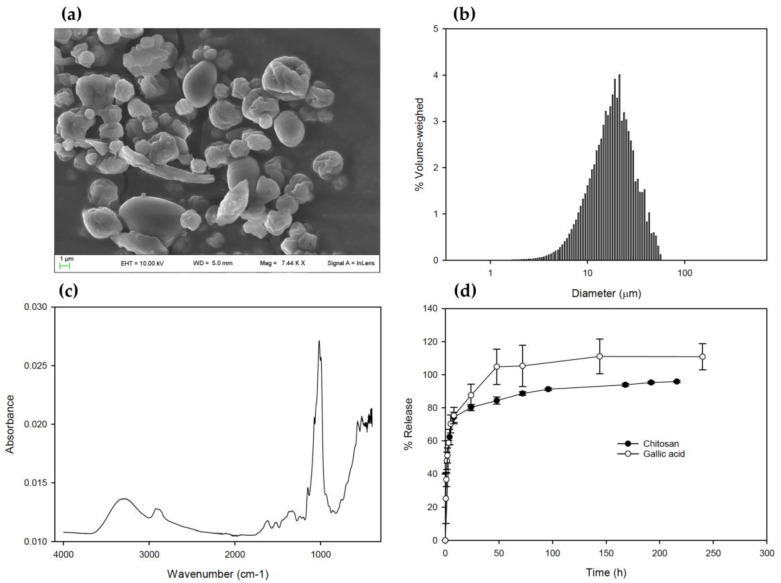
Characterization of the GA and CH loaded spray-dried nanostructured particles: (**a**) SEM picture obtained at 10 kV and 7 kX magnification, bar 1 µm; (**b**) volume-weighed particle size distribution; (**c**) ATR-IR profile; (**d**) in vitro release profiles of GA and CH from the particles performed at room temperature in water.

The obtained particles were not homogeneous in shape with some partially buckled structures, which typically occurs in spray-dried powders, and partially corrugated as well (Figure 2a). Volumetric size distribution was slightly broad and coherent with SEM observations with an average volume diameter of about 20 µm (Figure 2b). The in vitro GA and CH release profiles displayed a burst release (Figure 2d), with 67% of GA released within the first 5 h and 83% within a day. After 48 h, GA release reached a plateau (93% of released GA). The remaining GA was slowly released up to 8 days. Similarly, CH reached 62% during the first 4 h. After 24 h, it plateaued to 80% and the remaining was released slowly up to 8 days. Overall, with the active compounds being fairly soluble, the main release mechanism was assumed as a combination of diffusion–dissolution-controlled processes with an expected initial burst and a successive slower release. The observed fast diffusion may be reduced in non-sink conditions as in a real-world on-field application.

### 2.3. The NPF Displayed Several In Vitro Antifungal Modes of Action

**Figure 3:**
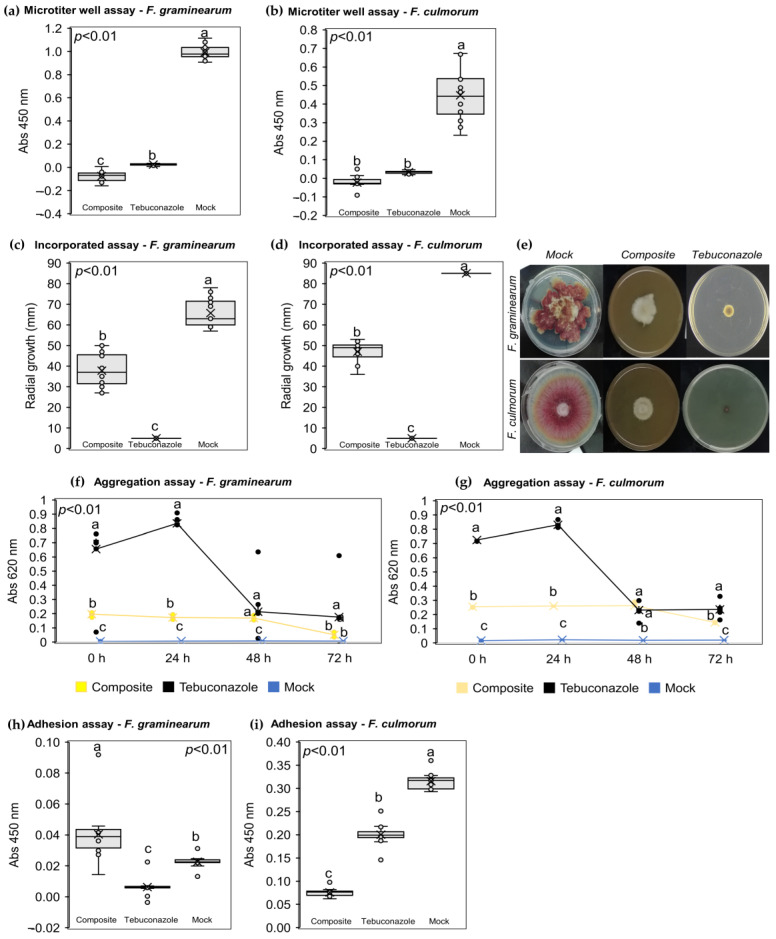
In vitro antifungal assays conducted against *F. graminearum* and *F. culmorum*. Boxplots of the in-broth microtiter well assay against *F. graminearum* (**a**) and *F. culmorum* (**b**) conidia; the number of germinated spores was determined by recording the mycelium absorbance at 450 nm at 48 h after incubation. Boxplots of the incorporated assay against *F. graminearum* (**c**) and *F. culmorum* (**d**) mycelium; the data were recorded as radial mycelium growth at 7 days post-incubation. Pictures of fungal colonies grown on PDA and PDA incorporated with the NPF and tebuconazole at 7 days post incubation (**e**). Boxplots of the aggregation assay against *F. graminearum* (**f**) and *F. culmorum* (**g**) conidia; the amount of aggregated conidia was determined by recording the conidia absorbance at 620 nm at 0, 24, 48, and 72 h post-incubation. Boxplots of the adhesion assay against *F. graminearum* (**h**) and *F. culmorum* (**i**) conidia; the amount of adhered conidia was determined by recording the mycelium absorbance at 450 nm at 24 h post incubation. The boxplots were obtained by plotting the data collected from three independent experiments, each one consisting of four replicates for each substance tested. Different letters (a, b, c) into the subfigures refer to the statistical analysis performed using one-way analysis of variance (ANOVA) with the Tukey test at a confidence level of 0.99 and *p* < 0.01.

The NPF displayed a direct antifungal activity against both *F. graminearum* and *F. culmorum* conidia and mycelium. Indeed, the NPF inhibited *F. graminearum* (Figure 3a) conidial germination (the average absorbance was around 0.00) compared to tebuconazole (average absorbance of 0.24) and mock control (average absorbance of 0.99). At the same time, the NPF inhibited *F. culmorum* (Figure 3b) conidial germination (average absorbance of 0.00) as well as tebuconazole (average absorbance of 0.033), while the average absorbance recorded on mock control was 0.447. Moreover, the NPF inhibited the *F. graminearum* (Figure 3c) and *F. culmorum* (Figure 3d) radial growth (37.83 mm and 47 mm, respectively) compared to the mock controls (65.75 mm and 85 mm, respectively), but not as much as tebuconazole (5 mm). Figure 3e shows the mycelium radial growth on Petri dishes under the different treatment conditions.

The NPF mechanically interacted with conidia, thus displaying indirect antifungal activity. The absorbances recorded during the aggregation assay revealed a similar mechanical interaction pattern in both *F. graminearum* (Figure 3f) and *F. culmorum* (Figure 3g). The mock controls displayed an average absorbance of 0.00, validating the correctness and accuracy of the assay, since the untreated conidia did not aggregate. At the same time, the NPF favored conidial flocculation compared to the mock controls but not as much as tebuconazole. Conidial flocculation favored by the NPF was quite stable among the different time points (the recorded average absorbances were 0.20–0.17–0.17–0.05 for *F. graminearum* and 0.26–0.26–0.26–0.15 for *F. culmorum*), while conidial flocculation favored by tebuconazole reached a plateau at 24 h and then decreased to the same levels recorded in the NPF treated samples (0.66–0.84–0.21–0.18 for *F. graminearum* and 0.73–0.83–0.23–0.24 for *F. culmorum*). The ability of the NPF to inhibit the conidia adhesion on a putative host tissue was also evaluated. The NPF did not inhibit *F. graminearum* conidia adhesion (Figure 3h) (the average recorded absorbance was 0.040) compared to tebuconazole (average recorded absorbance of 0.006) and mock control (average recorded absorbance of 0.023). On the other hand, the NPF significantly inhibited *F. culmorum* adhesion assay (Figure 3i) (the average recorded absorbance was 0.076) compared to tebuconazole (average recorded absorbance was 0.200) and mock control (the average recorded absorbance was of 0.316).

### 2.4. The NPF Displayed Biocompatibility on Bread Wheat Plants

Coating the kernels with the NPF did not affect germination in Sumai3 and Cadenza, while a reduction in kernel germination of Cadenza SBEIIa was observed, where 90.66% of kernels were germinated compared to mock control (100%) (Figure S1a). NBI (Figure S1b) and dry biomass (Figure S1c) on the three bread wheat genotypes were not affected; thus, the NPF did not show phytotoxicity on wheat plants.

### 2.5. The NPF Reduced FHB and FCR Symptoms Progression

**Figure 4:**
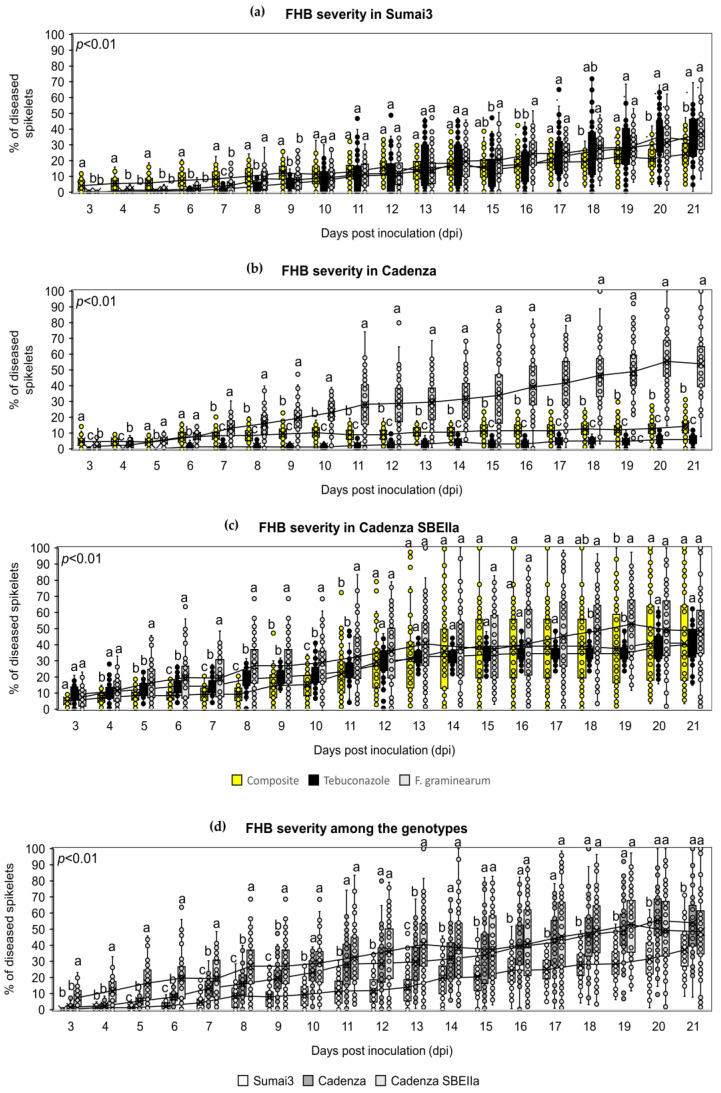
In planta FHB antifungal assays. FHB severity was monitored from 3 to 21 days post inoculation (dpi) by visually counting the diseased spikelets on the total spikelets for each spike. Boxplots of FHB severity in Sumai3 (**a**), Cadenza (**b**), Cadenza SBEIIa (**c**) treated with the NPF, tebuconazole, and solely inoculated spikes. Boxplots of FHB severity among the genotypes (**d**) solely inoculated. The boxplots were obtained by plotting the data collected from three independent experiments, each one consisting of twenty spikes for each experimental group. Different letters (a, b, c) into the subfigures refer to the statistical analysis performed using one-way analysis of variance (ANOVA) with the Tukey test at a confidence level of 0.99 and *p* < 0.01.

The preventive application of the NPF on bread wheat spikes reduced FHB symptoms in Sumai3 and Cadenza at the end of the infection trial (21 days post-inoculation, dpi). At 21 dpi, the average FHB severity was 24.11% in Sumai3 treated with the NPF compared to tebuconazole-treated plants (32.68%) and solely inoculated plants (37.17%) (Figure 4a and Figure S2). A similar pattern was observed in Cadenza, where at 21 dpi a drastic reduction in FHB symptoms development was recorded. Indeed, at 21 dpi, the average FHB severity was 14.25% in Cadenza treated with the NPF compared to tebuconazole-treated plants (5.79%) and solely inoculated plants (54.13%) (Figure 4b and Figure S2). On the other hand, an FHB symptoms reduction was observed from 4 to 11 dpi in Cadenza SBEIIa treated with the NPF, while this antifungal effect was completely lost at the end of the infection trial. Indeed, at 21 dpi, the average FHB severity was 40.98% in Cadenza SBEIIa plants treated with the NPF compared to tebuconazole (40.37%) and solely inoculated plants (48.39%) (Figure 4c and Figure S2). In order to evaluate the FHB response of Cadenza and Cadenza SBEIIa, the solely inoculated plants from the three bread wheat genotypes were compared (Figure 4d). From 3 to 9 dpi, FHB symptoms progression was lower in Cadenza compared to Cadenza At 21 dpi, the average FHB severity was of 37.17% in Sumai3, 54.13% in Cadenza, and 48.39% in Cadenza SBEIIa.

**Figure 5:**
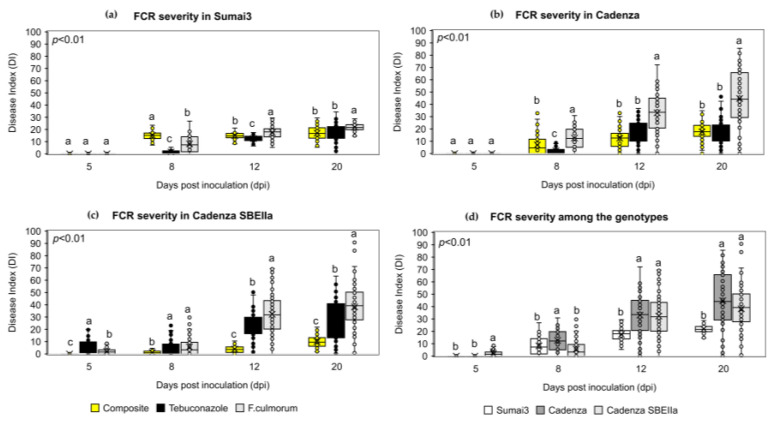
In planta FCR antifungal assays. FCR severity was monitored at 5, 8, 12, and 20 days post-inoculation (dpi). Boxplots of FCR disease index in Sumai3 (**a**), Cadenza (**b**), Cadenza SBEIIa (**c**) treated with the NPF, tebuconazole, and solely inoculated plants. Boxplots of FCR severity among the genotypes (**d**) solely inoculated. The boxplots were obtained by plotting the data collected from three independent experiments, each one consisting of twenty plantlets for each experimental group. Different letters (a, b, c) into the subfigures refer to the statistical analysis performed using one-way analysis of variance (ANOVA) with the Tukey test at a confidence level of 0.99 and *p* < 0.01.

The preventive application of the NPF on bread wheat spikes reduced FCR symptoms in the three bread wheat genotypes. In Sumai3 (Figure 5a and Figure S2), the application of the NPF reduced FCR symptoms development at 12 and 20 dpi. Indeed, at 20 dpi, the average FCR disease index was 17.01 in the NPF-treated plants, 18.04 in tebuconazole-treated plants, and 21.86 in the solely inoculated plants. A similar behavior was observed in Cadenza (Figure 5b and Figure S2), where at 20 dpi, the average FCR disease index was 17.62 in the NPF-treated plants, 16.67 in the tebuconazole-treated plants, and 44.85 in the solely inoculated plants. Surprisingly, the application of the NPF was particularly efficient in Cadenza SBEIIa (Figure 5c and Figure S2), where the FCR symptoms reduction was observed from 5 to 20 dpi. Notably, the application of the NPF was more effective than tebuconazole in controlling FCR progression. At 20 dpi, the average FCR disease index was 10.06 in the NFP-treated plants, 29.29 in the tebuconazole-treated plants, and 38.26 in the solely inoculated plants. In order to evaluate the FCR response of Cadenza and Cadenza SBEIIa, the solely inoculated plants of the three bread wheat genotypes were compared. FCR symptoms in the three bread wheat genotypes were similar at 5 and 8 dpi, while at 12 and 20 dpi, Sumai3 was phenotypically resistant to FCR compared to the two Cadenza genotypes. At 20 dpi, the FCR disease index was 21.86 in Sumai3, 44.85 in Cadenza, and 38.26 in Cadenza SBEIIa (Figure 5d).

### 2.6. The NPF Acts as an Elicitor-like Molecule by Greatly Boosting Innate Immunity in Cadenza SBEIIa

The expression level of twenty-one genes involved in the induction of innate immunity in bread wheat was evaluated 48 h after treatment or inoculation. Figure S3a shows the amplification of *TaICS*, *TaCHI*, *TaLOX*, *TaWRKY*, *TaPR5*, *TaNPR1*, *TaPDF*, *TaPR10*, *TaPR12*, *TaPR14*, *TaPEN1*, *TaFMO1*, *TaLLP1*, and *TaCAMTA3*, for which the primer pairs were tested in this work, while primer pairs for *TaCDPK*, *TaRBOH*, *TaPAL*, *TaMAPK*, *TaPR1*, *TaAOS*, and *TaPR2* have already been tested in previous literature, showing also the successful amplifications [19,20,21]. Figure S3b shows *TaACT* amplification from cDNA from Sumai3, Cadenza, and Cadenza SBEIIa, demonstrating that the cDNA was successfully synthesized and amplifiable.

**Figure 6:**
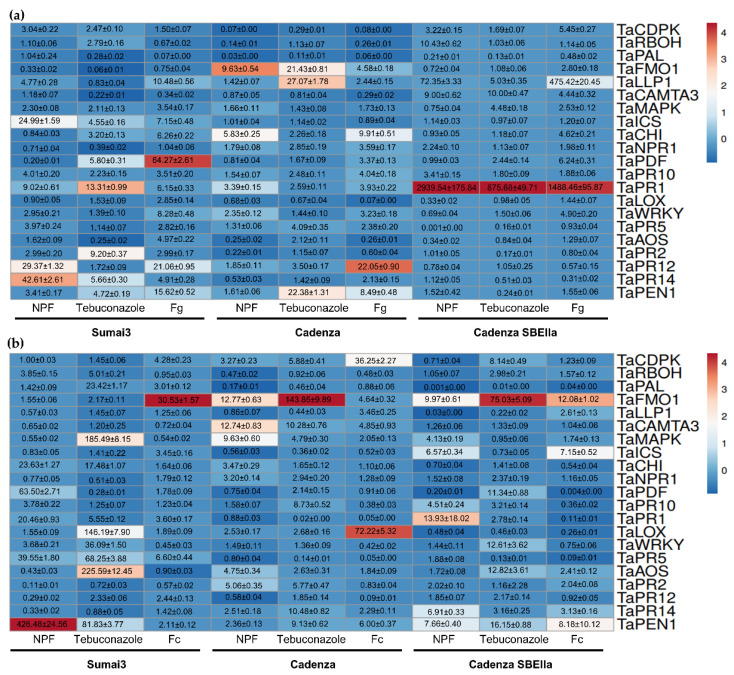
Heatmaps, relative expression levels, and standard deviations of the selected bread wheat genes in Sumai3, Cadenza, and Cadenza SBEIIa in the NPF and tebuconazole treated and the solely *F. graminearum*-inoculated spikes (**a**) and solely *F. culmorum*-inoculated plantlets (**b**). Relative expression values were obtained by using the equation 2^−ΔΔCq^ with *TaFNR*, *TaTUB*, and *TaACT* as reference genes and mock treatment used to normalize the relative expression levels. The heatmaps were constructed with the z-score (explained by the color legend at the left of the picture) by analyzing data with ClustVis software (Tartu, Estonia).

The expression pattern of the analyzed genes in wheat heads treated with the NPF or tebuconazole or solely inoculated with *F. graminearum* (Figure 6a) revealed that *TaICS* (24.90-fold), *TaPR12* (29.37-fold), and *TaPR14* (42.61-fold) were strongly overexpressed in the NPF heads compared to tebuconazole (4.55-fold, 1.72-fold, and 5.66-fold, respectively) and solely inoculated plants (7.15-fold, 21.06-fold, and 4.91-fold), while the other genes were basal or slightly up-regulated. *F. graminearum* strongly up-regulated *TaPDF* (64.27-fold), while it was down-regulated in the NPF-treated plants (0.20-fold). In Cadenza, *TaFMO1* was the most up-regulated gene (9.63-fold) in the NPF spikes compared to the solely inoculated plants (4.58-fold). A strong down-regulation of *TaCDPK* and *TaPAL* was observed in both treated (0.07-fold and 0.03-fold) and solely inoculated heads (0.08-fold and 0.06-fold). Tebuconazole up-regulated *TaFMO1* (21.43-fold), *TaLLP1* (27.07-fold), and *TaPEN1* (22.38-fold), while *TaPR12* was up-regulated by *F. graminearum* (22.05-fold). Despite Cadenza SBEIIA being the most susceptible genotype from the phenotypic evaluation of FHB symptoms, the expression pattern that resulted was extremely interesting, since some genes were strongly up-regulated in the NPF-treated heads. *TaPR1* was the most up-regulated gene (2939.54-fold), followed by *TaLLP1* (72.35-fold), *TaRBOH* (10.43-fold), *TaCAMTA3* (9.00-fold), *TaPR10* (3.41-fold), *TaCDPK* (3.22-fold), and *TaNPR1* (2.40-fold). *TaPR5* was strongly down-regulated (0.001-fold), while the other genes were mainly slightly down-regulated or basally expressed. *TaPR1* was the only gene strongly up-regulated in the tebuconazole-treated spikes (875.68-fold), followed by *TaCAMTA3* (10.00-fold), *TaLLP1* (5.03-fold), and *TaMAPK* (4.48-fold), while the remaining genes were mostly basal or down-regulated. In the solely inoculated plants, *TaPR1* (1488.45-fold) and *TaLLP1* (475.42) were strongly up-regulated, followed by *TaPDF* (6.24-fold), *TaCDPK* (5.45-fold), *TaWRKY* (4.90-fold), *TaCHI* (4.62-fold), and *TaCAMTA3* (4.44-fold), while the remaining genes were basal or down-regulated. 

The expression pattern of the analyzed genes in wheat plantlets treated with the NPF or tebuconazole or solely inoculated with *F. culmorum* (Figure 6b) revealed that in the NPF-treated Sumai3 plantlets, *TaPEN1* (426.48-fold) was strongly up-regulated, followed by *TaPDF* (63.50-fold), *TaPR5* (39.55-fold), *TaCHI* (23.63-fold), and *TaPR1* (20.46-fold). In the tebuconazole-treated plantlets, *TaAOS* (225.60-fold), *TaMAPK* (185.49-fold), *TaLOX* (146.19-fold), *TaPEN1* (81.83-fold), *TaPR5* (68.25-fold), *TaWRKY* (36.09-fold), *TaPAL* (23.42-fold), and *TaCHI* (17.48-fold) were strongly up-regulated, while the remaining genes were mostly basal or slightly down-regulated. In the solely inoculated plants, only *TaAOS* (79.32-fold) and *TaFMO1* (30.53-fold) were strongly up-regulated. In Cadenza, the application of the NPF up-regulated *TaFMO1* (12.77-fold), *TaCAMTA3* (12.74-fold), and *TaMAPK* (9.63-fold), while the other genes were slightly up-regulated or down-regulated. Tebuconazole treatment strongly up-regulated *TaFMO1* (143.85-fold), while strongly down-regulated *TaPR1* (0.02-fold). Inoculation with *F. culmorum* strongly up-regulated *TaLOX* (72.22-fold) and *TaCDPK* (36.25-fold), while strongly down-regulated *TaPR1* (0.05-fold), *TaPR5* (0.05-fold), and *TaPR12* (0.09-fold). In Cadenza SBEIIa, the NPF up-regulated *TaPR1* (13.93-fold), *TaFMO1* (9.97-fold), *TaPEN1* (7.66-fold), *TaPR14* (6.91-fold), *TaICS* (6.57-fold), *TaPR10* (4.52-fold), and *TaMAPK* (4.13-fold), while strongly down-regulated *TaPAL* (0.001-fold) and *TaLLP1* (0.03-fold). Tebuconazole strongly induced the expression of *TaFMO1* (75.03-fold), while down-regulated *TaPAL* (0.01-fold). Inoculation with *F. culmorum* induced the expression of *TaFMO1* (12.08-fold) and *TaICS* (7.15-fold), while strongly down-regulated *TaPAL* (0.04-fold), *TaPDF* (0.004-fold), and *TaPR5* (0.09-fold). 

### 2.7. The NPF Increased Grain Yield in the Two Cadenza Genotypes while Decreasing the Fusarium graminearum Biomass Spread into the Spikes

**Figure 7:**
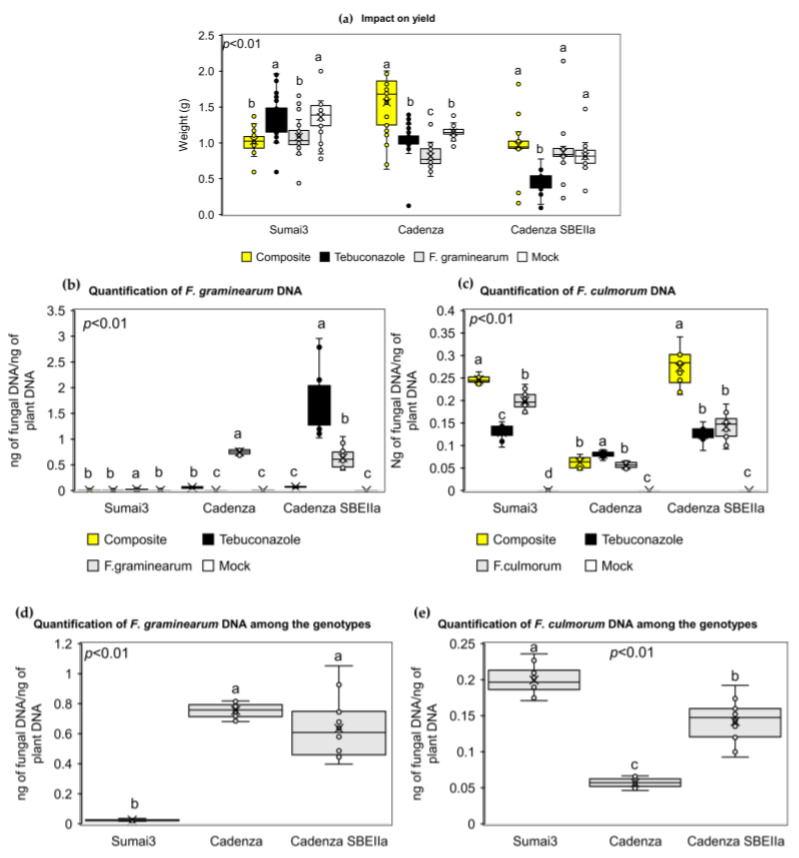
Impact on yield in Sumai3, Cadenza, and Cadenza SBEIIa after preventively treating the spikes with the NPF, tebuconazole, solely *F. graminearum*-inoculated plants and untreated and uninoculated spikes as mock control (**a**). *F. graminearum* (**b**) and *F. culmorum* (**c**) biomass quantification expressed as ng of fungal DNA/ng of plant DNA from Sumai3, Cadenza, and Cadenza SBEIIa spikes or plantlets preventively treated with the NPF, tebuconazole, solely *F. graminearum* or *F. culmorum*-inoculated plants and untreated and uninoculated plants as mock control. Quantification of *F. graminearum* (**d**) and *F. culmorum* (**e**) into the solely inoculated plants to compare the putative ability of the different genotypes to counteract fungal spread. The boxplots were obtained by plotting the data collected from three independent experiments, each one consisting of twenty spikes for each experimental group. Different letters (a, b, c) into the subfigures refer to the statistical analysis performed using one-way analysis of variance (ANOVA) with the Tukey test at a confidence level of 0.99 and *p* < 0.01.

The application of the NPF on the spikes of the three genotypes had a different behavior on the final yield. A significant reduction was observed in Sumai3 solely inoculated (average weight of 1.08 g) plants and those with the NPF (average weight of 1.03 g); differently, the application of tebuconazole showed a yield (average weight of 1.33 g) comparable with the mock controls (average weight of 1.36 g). On the other hand, in the two Cadenza genotypes, the application of the NPF increased the final yield. Indeed, in Cadenza the average final weight was of 1.56 g, 1.04 g, 0.81 g, and 1.15 g in the NPF, tebuconazole, solely inoculated and mock plants, respectively. In Cadenza SBEIIa, the final average weight was of 0.97 g, 0.46 g, 0.87 g, and 0.81 g in the NPF, tebuconazole, solely inoculated and mock plants, respectively (Figure 7a).

Quantification of fungal biomass was performed by Real-Time qPCR. Figure S4 shows the standard curves developed in this study for the amplification and quantification of TaACT in Sumai3, Cadenza, and Cadenza SBEIIa (Figure S4a–c) and for the amplification and quantification of Tri5 in *F. culmorum* (Figure S4d). The standard curve for the quantification of Tri6 in *F. graminearum* was already validated in a previous work [22]. Reaction efficiency (E) ranged from 1.16 to 1.65, while R^2^ ranged from 0.94 to 0.99. The quantification of fungal biomass indicated that the preventive application of the NPF on Sumai3 heads reduced pathogen spread as much as tebuconazole and the mock controls. Indeed, the same amount of ng of fungal DNA/ng of plant DNA was detected in the NPF, tebuconazole, and mock plants (0.01 ng of fungal DNA/ng of plant DNA) compared to solely *F. graminearum*-inoculated heads (0.03 ng of fungal DNA/ng of plant DNA). In Cadenza, the preventive application of the NPF reduced the amount of the accumulated fungal biomass (0.06 ng of fungal DNA/ng of plant DNA) compared to the solely *F. graminearum*-inoculated spikes (0.76 ng of fungal DNA/ng of plant DNA). The application of tebuconazole was more efficient (0.01 ng of fungal DNA/ng of plant DNA) in the prevention of fungal infection and was comparable to mock control plants (0.01 ng of fungal DNA/ng of plant DNA). On the other hand, the preventive application of the NPF in Cadenza SBEIIa drastically reduced the amount of the accumulated fungal biomass (0.07 ng of fungal DNA/ng of plant DNA), which was statistically similar to mock control plants (0.003 ng of fungal DNA/ng of plant DNA), compared to the solely *F. graminearum*-inoculated spikes (0.64 ng of fungal DNA/ng of plant DNA). Surprisingly, the preventive application of tebuconazole in Cadenza SBEIIa favored *F. graminearum* spread (1.77 ng of fungal DNA/ng of plant DNA) (Figure 7b). A dissimilar behavior was observed in the *F. culmorum* quantification in bread wheat plantlets. The preventive application of the NPF in Sumai3 was not effective in controlling fungal spread (0.25 ng of fungal DNA/ng of plant DNA) compared to tebuconazole (0.13 ng of fungal DNA/ng of plant DNA) and solely *F. culmorum*-inoculated plantlets (0.20 ng of fungal DNA/ng of plant DNA), while no DNA was detected in mock control plants. In Cadenza, the accumulated fungal biomass in the NPF-treated plantlets was similar to the solely *F. culmorum*-inoculated plantlets (0.06 and 0.06 ng of fungal DNA/ng of plant DNA, respectively), while tebuconazole favored the accumulation of fungal biomass (0.08 ng of fungal DNA/ng of plant DNA), while no fungal DNA was detected in mock control plants. In Cadenza SBEIIa, the preventive application of the NPF favored the accumulation of *F. culmorum* (0.27 ng of fungal DNA/ng of plant DNA) compared to tebuconazole (0.12 ng of fungal DNA/ng of plant DNA) and solely *F. culmorum*-inoculated plantlets (0.14 ng of fungal DNA/ng of plant DNA), while no fungal DNA was detected in mock control plants (Figure 7c). Moreover, the data obtained from the solely inoculated spikes and plantlets were compared in order to evaluate the FHB and FCR response in the three bread wheat genotypes. Figure 7d confirms the strong effect of the FHB type II resistance in Sumai3 (0.03 ng of fungal DNA/ng of plant DNA), while no type II resistance was detected in Cadenza and Cadenza SBEIIa (0.76 and 0.64 ng of fungal DNA/ng of plant DNA, respectively). On the other hand, Figure 7e shows that Sumai3 does not possess the ability to counteract *F. culmorum* spread (0.20 ng of fungal DNA/ng of plant DNA), while Cadenza SBEIIa demonstrated tolerance and Cadenza was resistant to *F. culmorum* spread (0.14 and 0.06 ng of fungal DNA/ng of plant DNA, respectively).

### 2.8. The Interaction among Data Revealed That the Preventive Application of the NPF Contributed to Control In Vivo FHB and FCR

Principal Component Analysis (PCA) was performed to evaluate the interaction among the single data obtained during the in vivo experiments, in order to assess if the application of the NPF was effectively able to control FHB and FCR. Figure S5a shows PCA carried out on FHB data. The data clearly clustered as “contributive” (in red) and “not contributive” (in blue) to FHB control. As expected, all the data derived from the mock control plants clustered as “contributive”, since the data were obtained from uninoculated spikes. Moreover, all the data obtained from Sumai3 clustered as “contributive”, since even in the solely *F. graminearum*-inoculated plants, the genotype showed FHB resistance. Importantly, all the data derived from the NPF-treated plants clustered as “contributive”, indicating that the preventive application of the NPF also contributes to FHB control in susceptible genotypes. Only the data derived from Cadenza SBEIIa treated with the tebuconazole and Cadenza or Cadenza SBEIIa solely inoculated clustered as “not contributive”, indicating that the application of the conventional fungicide tebuconazole is not able to efficiently control FHB in a very susceptible genotype. Figure S5b shows PCA carried out on FCR data. In this case, the data did not clearly cluster as in Figure S5a, since a partial overlap is present. As expected, all the data derived from mock plants clearly clustered together as “contributive” and separated from the rest of the data. The preventive application of the NPF in Cadenza and of tebuconazole in Sumai3 and Cadenza clearly contributed to FCR control, while data obtained from Cadenza SBEIIa treated with the NPF clustered borderline. Data from the solely inoculated Cadenza and Cadenza SBEIIa genotypes clustered as “not contributive”, while data from Sumai3 treated with the NPF, Sumai3 solely inoculated, and SBEIIa treated with tebuconazole overlapped.

### 2.9. The NPF Application Affects the Expression Levels of Starch Biosynthetic Genes and Total Starch Content in Grains

**Figure 8:**
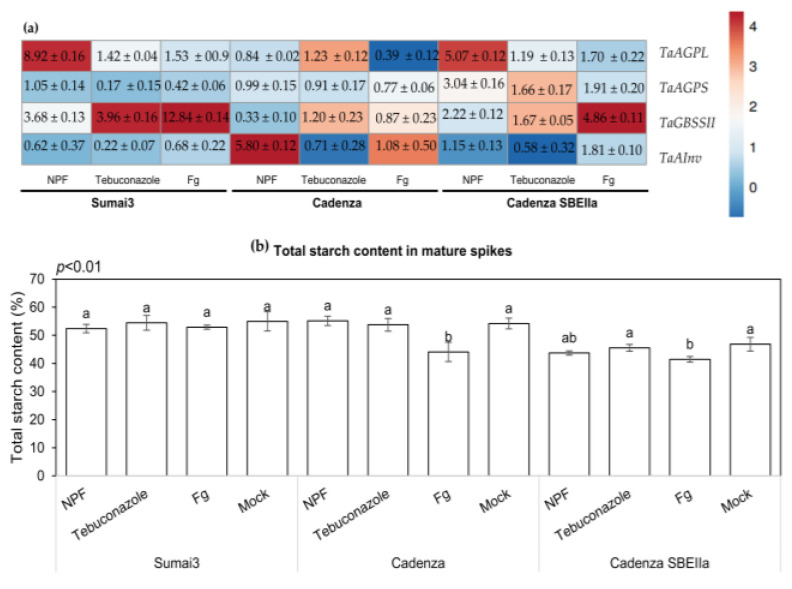
Heatmap, relative expression levels, and standard deviations of the selected genes involved in transitory starch biosynthesis in Sumai3, Cadenza, and Cadenza SBEIIa inoculated with *F. graminearum* and treated with NPF or tebuconazole. Relative expression values were obtained by using the equation 2^−ΔΔCq^ with *TaACT* as reference genes and mock treatment used to normalize the relative expression levels. The heatmaps were constructed with the z-score (explained by the color legend at the left of the picture) by analyzing data with ClustVis software (Tartu, Estonia) (**a**). Total starch content (%) determined in mature spikes of Sumai3, Cadenza, and Cadenza SBEIIa subjected to the different treatments. The averages and standard deviations were calculated from three biological replicates, each one constituted a bulk of 10 spikes. Different letters (a, b) into the subfigures refer to the statistical analysis performed using one-way analysis of variance (ANOVA) with the Tukey test at a confidence level of 0.99 and *p* < 0.01 (**b**).

The expression level of four genes involved in the transitory starch biosynthesis in wheat was evaluated at 48 h after inoculation with *F. graminearum* and treatment with the NPF or tebuconazole. Inoculation with *F. graminearum* in Sumai3 up-regulated *TaGBSSII* (8.92-fold) and *TaAGPL* (1.53-fold) and down-regulated *TaAGPS* (0.42-fold). A different behavior was observed in Cadenza: *F. graminearum* inoculation down-regulated *TaAGPL* (0.39-fold) and did not affect *TaGBSSII*, *TaAGPS*, and *TaAInv*. Instead, all the analyzed genes were up-regulated in the solely inoculated plants of Cadenza SBEIIa. The NPF treatment in Sumai3 and Cadenza SBEIIa up-regulated *TaAGPL* (8.92-fold and 5.07-fold, respectively) and *TaGBSII* (3.68-fold and 2.22-fold, respectively). On the other hand, the NPF treatment resulted in a different effect in Cadenza: it down-regulated *TaGBSSII* (0.33-fold) and up-regulated *TaAInv* (5.80-fold). Tebuconazole treatment did not significantly affect *TaAGPL* in all the genotypes, whereas up-regulated *TaGBSSII* and decreased *TaAGPS* and *TaAInv* (Figure 8a).

The total starch content was also evaluated in the harvested mature kernels of the three wheat genotypes subjected to *F. graminearum* inoculation, NPF, or tebuconazole-treated and mock plants. The total starch content in Sumai3 was not affected by inoculation or treatments compared to the mock control. In Cadenza and Cadenza SBEIIa, a significant reduction in starch content was observed in the solely inoculated plants. Additionally, the starch content was similar in Cadenza treated with NPF, tebuconazole, and mock plants (Figure 8b).

## 3. Discussion

In the present research work, we synthesized and tested for its antifungal and elicitor-like properties a novel NPF formed of CNC as carriers, HAS as excipient, and CH and GA as active compounds. CNC and other cellulose-derived polymers have already been employed and studied for their powerful application in the composition of novel agrochemicals for disease management [23]. Some examples reported that CNC have been evaluated as a possible nanostructured formulation to be directly applied as plant protection treatments on tomato plants to control the bacterial pathogen *Pseudomonas syringae* pv. *tomato* (Pst), the causal agent of bacterial speck disease. CNC applied on tomato plants reduced Pst epiphytic survival, thus diminishing the amount of the starting inoculum [24]. In another research work, CNC was synthesized by cellulose extracted from *Actinidia deliciosa* pruning residues and functionalized as carriers to vehicle carvacrol as an active antimicrobial, in order to manage several plant bacterial diseases. CNC as carriers modulated the carvacrol mechanical and chemical responses and enhanced its antimicrobial properties against microorganism contamination [25]. CNC have also been employed as antimicrobials against *Pseudomonas savastanoi* pv. *savastanoi* on olive plants. CNC employed at 1% in aqueous solution inhibited in vitro bacterial growth and biofilm formation, while on plants they reduced Psav epiphytic survival and demonstrated biocompatibility on olive leaves. Moreover, the study indicated that CNC are uptaken at the root level, indicating that CNC could be also used as a carrier system for active compounds [26]. Finally, a recent study employed ethyl cellulose-based nano-delivery for the encapsulation of phenamacril to improve the control of FHB. The encapsulated phenamacril showed excellent storage stability and wettability on leaves, while showing improved antifungal properties compared to the commercial preparation in controlling *F. graminearum* [27]. Starch was employed as an excipient to increase water solubility and the slow release of active principles. As an example, neem seed oil was encapsulated in urea-formaldehyde-crosslinked starch and guar gum for the formation of a natural liquid pesticide and tested for its physical and chemical properties [28]. More recently, a research work employed a composite formed with CNC, standard starch, or HAS as excipient, and CH as the antibacterial compound, to control Pst on tomato plants. The authors suggest that CNC and starch helped in the CH-controlled release, while diminishing Pst epiphytic survival and being completely biocompatible on tomato plants [18]. These data are in line with those obtained in the present study since CNC and HAS were suitable as carriers for active compounds and excipients, while optimizing their controlled release. Moreover, to our knowledge, this is the first research work employing CNC and HAS to formulate a novel agrochemical for the management of FHB and FCR. In the present study, CH and GA were used as antifungal molecules. CH and its derivates, such as CH nanoparticles, or chitin were also tested for their antifungal properties against *F. graminearum* and *F. culmorum*. Kheiri et al. demonstrated that CH and CH nanoparticles inhibited *F. graminearum* radial mycelial growth as well as reduced the disease severity in artificially inoculated plants [29,30]. Another study combined CH and a liquid seaweed extract (LSE). Both CH used alone and combined with LSE demonstrated in vitro antifungal activity, reduced the necrotic area on leaves and up-regulated *TaPR1* and *TaPR2* in wheat seedlings [31]. More recent studies confirmed the antifungal activity of CH against *F. graminearum*, since even low CH concentrations inhibited fungal growth rates as well as the amount of accumulated DON and fumonisin in wheat grains [32]. Similar results have been also obtained in durum wheat, where the preventive application of CH on wheat heads or flag leaves on a susceptible and a resistant genotype decreased FHB symptoms while boosting innate immunity by the up-regulation of *TaPR1*, *TaPR2,* and *TaPAL*. Interestingly, the same study also evaluated the expression level of the main genes involved in *F. graminearum* virulence and trichothecenes biosynthesis, and a strong down-regulation was observed when treating *F. graminearum* with CH, while the trichothecene biosynthetic pathway was up-regulated when treating *F. graminearum* with commercial fungicides. This was also confirmed by detecting and quantifying the mycotoxins in infected grains, where increased amounts of DON were detected in the samples subjected to the commercial formulations treatment [19]. CH oligomers have also been conjugated with amino acids and tested for their antifungal properties against *F. culmorum*. The conjugated complexes demonstrated in vitro antifungal activity and the most promising one that resulted was CH conjugated with tyrosine, which was assayed on field trials applied on *T. spelta*, where the DON content was drastically reduced [33]. Other authors have investigated the antifungal mode of action of CH polymers and oligomers by observing *F. graminearum* morphology after being in contact with CH. These authors observed a delay in germination and in the elongation of germ tubes and an alteration of hyphal morphology [34]. Finally, CH hydrochloride showed in vivo antifungal ability in the FHB-susceptible wheat genotype Remus and its preventive application also positively affected grain yield. Moreover, the metabolomic approach revealed the up-regulation of jasmonic acid-related metabolites, which are linked to the FHB resistance pathway in wheat [35]. In the present study, GA was coupled with CH as an active compound against FHB and FCR. GA is a phenolic compound, a class of molecules that seems to play a prominent role in wheat resistance against *Fusarium* diseases, since higher levels of phenolic compounds have been detected in resistant wheat cultivars compared to susceptible ones after inoculation with different *Fusarium* spp. [36,37]. This evidence suggests that the exogenous application of phenolic compounds can act as elicitors of innate immunity. As an example, a natural wheat bran extract was added to *F. culmorum* liquid cultures, in order to monitor trichothecene biosynthesis. It was observed that most of the trichothecene biosynthetic genes were drastically down-regulated, resulting in a sharp reduction in type B trichothecene, which was related to the phenolic compounds present in the bran extract [38]. Another study characterized the phenolic compounds in a culture extract of *Spirulina* algae and applied it in vitro against *F. graminearum*. The extract was mainly composed of GA and was extremely efficient in inhibiting fungal growth as well as trichothecene production [39,40]. GA and other phenolic compounds were also conjugated to silver nanoparticles in order to enhance their antifungal ability against *F. culmorum* [41]. A more recent study investigated the possible antifungal mechanism of Chinese galls extract, GA, and tannic acid against *F. graminearum*. The authors observed that such compounds reduced both conidia and ascospores germination as well as perithecia formation, thus inhibiting the first stage of fungal infection [42]. All this evidence regarding the antifungal properties of CH and GA is in agreement with those observed in the present work, since we reported a direct antifungal ability by in vitro experiments against *F. graminearum* and *F. culmorum*.

Among the different antifungal modes of action, we demonstrated that the NPF showed direct toxicity on *F. graminearum* and *F. culmorum*, but also a mechanical interaction with fungal cells by inhibiting cell aggregation and adhesion. The anti-aggregative and anti-adhesive strategies may enable new plant disease control methods that are environmentally compatible, because they do not necessarily require any uptake of compounds into the fungal cells or disruption of the eukaryotic metabolic pathway [43]. Little is known about the molecular structures responsible for cell aggregation and adhesion in *Fusarium* spp. Probably, adhesin-like proteins may be responsible for these mechanisms during the early stages of pathogenesis [44]. In the present research work, we observed that the application of the NPF can favor cell aggregation at a low level, while inhibiting cell adhesion in *F. culmorum,* but not in *F. graminearum*. The low level of cell aggregation and inhibition of cell adhesion may be attributed to the presence of CNC in the NPF. Indeed, CNC have already been investigated for their ability to favor bacterial aggregation and to inhibit bacterial adhesion in *Pseudomonas fluorescens* and *Escherichia coli*. The authors hypothesized that CNC mechanically interact with bacterial cells by a depletion mechanism, since they measured variations in the zeta potential of bacterial cells after treating them with CNC. Moreover, fluorescence microscopy clearly co-localized CNC on the *E. coli* surface, thus confirming the mechanical interaction of CNC with bacterial cells [45,46,47]. GA was also evaluated for its ability to inhibit bacterial cell adhesion and biofilm formation. Indeed, GA inhibited bacterial adhesion in *Streptococcus thermophilus*, *Staphylococcus aureus,* and *Xylella fastidiosa* [48,49]. To our knowledge, there is no evidence regarding the interactions between HAS or CH with fungal cells as anti-aggregative and anti-adhesive molecules; thus, such mechanisms need to be deeply investigated in order to reveal novel antifungal modes of action. Moreover, the mechanical interactions of CNC have only been investigated in relation to human bacterial or plant pathogens, while no direct evidence regarding fungal pathogens is available. Thus, the present research work is the first to highlight a mechanical interaction between the NPF and fungal cells as an indirect antifungal mode of action, which will result in a promising sustainable strategy for FHB and FCR management.

In the present research work, we demonstrated that the NPF was biocompatible on the three wheat genotypes since it did not negatively affect germination, NBI, and dry biomass. Only in Cadenza SBEIIa did we observe a slight germination reduction. To our knowledge, there is no evidence regarding the phytotoxicity of the exogenous application of CNC or HAS on plants, since they are glucose-based plant-derived polymers. CH has already been employed to boost plant growth and development and has been demonstrated to be biocompatible on plants, since its conjugation with fungicides was useful to decrease fungicide-associated phytotoxicity [18,19,50]. On the other hand, some research works reported that GA may demonstrate allelopathic properties, by causing phytotoxicity at different levels depending on its concentration and on the plant species in which GA is exogenously applied [51,52]. We may suppose that the reduction in kernel germination in Cadenza SBEIIa could be due to differences at the genomic level leading to a diverse response or metabolic pathway with respect to GA. However, further elucidations are needed to investigate the putative presence of unintended mutations involved in the response to the NPF.

As mentioned before, there are many research papers reporting the in vivo antifungal ability of CH and GA, as well as composites or complexes derived from these molecules. This evidence is in line with that observed in the present research work, since we observed a decrease in FHB symptoms in Sumai3 and Cadenza and a decrease in FCR symptoms in Sumai3. Interestingly, we did not observe a significant decrease in FHB symptoms in Cadenza SBEIIa at the end of the infection trial. Nevertheless, we detected a strong up-regulation of most of the genes involved in innate immunity in the Cadenza SBEIIa spikes treated with the NPF, resulting in an efficient containment of the spread of the fungal biomass compared to the tebuconazole-treated spikes and the solely inoculated spikes. Moreover, the final grain yield was also positively restored by the application of the NPF, despite FHB symptoms scoring high in the treated spikes. Such evidence clearly seems to be in contrast with what we observed during the phenotyping scoring of FHB symptoms. Actually, these contrasting results could have many explanations. The rapid bleaching of spikes, but low amounts of fungal DNA in Cadenza SBEIIa treated with the NPF, could be explained by the presence of a putative type II tolerance (defined as the ability to counteract fungal spread) in Cadenza SBEIIa, resulting in a blockage of plant vessels and preventing water and nutrient supplies as a defense mechanism, thus causing sudden spike wilting [53,54]. Indeed, spike bleaching due to a natural absence of water and nutrients could be confused with FHB symptoms [55]. This type II tolerance can also explain the reactiveness of some genes involved in innate immunity observed in Cadenza SBEIIa after the NPF treatments. To our knowledge, only waxy wheat genotypes (characterized by starch composed of high amylopectin content) have been investigated for FHB resistance [56,57]. Such genotypes have extremely variable FHB responses, demonstrating susceptibility or tolerance, depending on the predominant *Fusarium* spp. in the inoculum and on weather conditions. Since we observed such interesting FHB tolerance in Cadenza SBEIIa, we may hypothesize that the genotypes could possess mutations, due to the mutagenesis approach employed to generate the mutant, related to the differences in FHB responses compared to the wild-type genotype Cadenza. Thus, it would be desirable to sequence the Cadenza SBEIIa genome and to individuate novel allele variants related to FHB responses. 

A different behavior has been observed regarding FCR responses: FCR symptoms scoring revealed that the preventive application of the NPF on plantlets decreased FCR symptoms in all the three wheat genotypes. Nevertheless, a few genes were up-regulated after the application of the NPF and no containment of fungal spread was observed. This evidence could be explained by several factors. As FHB resistance, like FCR resistance, in wheat, is quantitative and affected by environmental factors, plant developments, and by the methods used to artificially inoculate and score the plants. Indeed, several studies and reviews have reported that the establishment of a glasshouse disease assay for the assessment of FCR resistance has been quite challenging and often not reproducible, by demonstrating performances differing from the ones observed in field conditions. A study reported a positive correlation between FCR symptoms scoring and fungal biomass accumulation in wheat, but the authors concluded that even if FCR symptoms correlate with the amount of fungal biomass, this measure is not a reliable indication of resistance or tolerance [58]. Moreover, in Sumai3, we did not observe a correlation between FCR symptoms scoring and fungal biomass quantification. Despite Sumai3 showing FCR resistance by symptoms scoring, it was not able to counteract fungal spread, and was more susceptible than Cadenza and Cadenza SBEIIa. As previously reported, FHB resistance is not correlated with FCR resistance in Sumai3, despite the two diseases being provoked by the same *Fusarium* spp. genus [58,59]. The data we obtained about the fungal accumulation in Sumai3, Cadenza, and Cadenza SBEIIa definitely need further investigations, since *F. culmorum* biomass quantification was never performed before, thus adding novel knowledge regarding FCR resistance in wheat. Moreover, since Cadenza and Cadenza SBEIIa demonstrated tolerance for counteracting *F. culmorum* spread, further studies are needed to understand if these genotypes may actually be a valuable source for FCR resistance. 

We evaluated the expression level of the main genes involved in innate immunity at 48 h after treatment or inoculation. Interestingly, a different expression pattern among the three bread wheat genotypes during the FHB assay was observed, but we also detected a strong up-regulation of some genes in Cadenza SBEIIa after treatment with the NPF. *TaPR1* was up-regulated in all the NPF-treated and *F. graminearum*-inoculated spikes, suggesting that both the treatment and the pathogen trigger a salicylic acid (SA)-mediated defense. This is also confirmed by the up-regulation of other SA-regulating genes in Sumai3 treated with the NPF and solely inoculated, such as *TaLLP1* (involved in the transmission of systemic acquired resistance, SAR, in systemic leaves), *TaICS* (involved in the SA biosynthesis from chorismate), *TaWRKY* (SAR positive regulator), and some PR-proteins such as *TaPR5*, *TaPR10,* and *TaPR12*. Interestingly, the up-regulation of *TaPDF*, *TaPEN1,* and *TaLOX* was only observed in the *F. graminearum*-inoculated spikes, suggesting that only the pathogen can trigger the jasmonic acid (JA)-mediate defenses and cellular apoptosis. In fact, *TaPDF* is activated by JA, *TaLOX* is involved in JA biosynthesis, and *TaPEN1* is activated by fungal penetration to further induce cell apoptosis. As previously mentioned, the preventive application of the NPF strongly up-regulated some genes in Cadenza SBEIIa, suggesting that this genotype may be particularly reactive to defense elicitors. *TaPR1*, *TaLLP1*, *TaRBOH* (involved in the oxidative burst), *TaCAMTA3* (involved in the elicitor-triggered immunity (ETI), and *TaPR10* trigger the defense responses in Cadenza SBEIIa, followed by the activation of *TaNPR1* (which induces the transcription of PR-protein genes in response to SA accumulation), strongly confirmed that the NPF induces the SA-mediated pathway. Indeed, the JA-mediated pathway (*TaPDF*, *TaAOS*, and *TaLOX*) is down-regulated. During the FCR experiments, the relative expression level of the investigated genes was extremely variable among genotypes; thus, it was not possible to individuate a putative pathway responsible for FCR resistance. Previous studies investigated the up-regulation of genes involved in innate immunity after the application of elicitor-like molecules. The application of CH was particularly studied as booster of transcription levels of PR genes, such as *TaPR1*, *TaPR2,* and *TaPR3* leading to SAR induction [19,31]. This is in line with our main findings, indicating that the SA-mediated defenses play a central role in elicitor-triggered innate immunity. Furthermore, we investigated the expression levels of 21 genes in total and most of them have been never evaluated for their role and responses toward FHB, FCR, or application of elicitor-like molecules. Thus, we believe that the present research only represents the first step toward a better understanding of the molecular mechanisms behind the challenging and practical employment of elicitor-like molecules to control FHB and FCR in wheat. Notably, some of the investigated genes were also up-regulated in spikes and plantlets treated with tebuconazole. To our knowledge, there are no transcriptomic approaches evaluating the elicitor-like activity of tebuconazole, but a research study investigating the effect of tebuconazole to control *Puccinia striiformis* on wheat reported that the application of tebuconazole enhanced structural and biochemical host defense reactions in the infected host, thus this evidence is in line with that observed in the present research work [60].

In the present study, we evaluated the expression levels of the genes involved in starch biosynthesis as well as the total starch content in mature kernels. The gene expression analysis revealed a similar behavior in Sumai3 and Cadenza SBEIIa, since infection with *F. graminearum* induced *TaAGPL* and *TaGBSSII* in both the genotypes, but not in Cadenza. Many transcriptional and proteomic studies highlighted that the starch biosynthetic pathway and starch content are negatively affected in FHB susceptible wheat genotypes, while starch biosynthesis is preserved in FHB-resistant genotypes [61,62]. The starch and sucrose metabolism pathway plays a critical role in FHB resistance, since FHB-resistant genotypes are capable of generating more energy and therefore could better respond to FHB. Particularly, a study observed that FHB-resistant genotypes accumulate alpha-amylase inhibitors, thus inhibiting amylases secreted by the pathogen and used to colonize kernels and acquire nitrogen and carbon from the endosperm [63,64]. The similar expression pattern observed in Sumai3 and Cadenza SBEIIa under *F. graminearum* infection suggests that the mutation responsible for HAS biosynthesis may positively affect the FHB response in Cadenza SBEIIa. Despite the starch biosynthetic pathway being elicited by *F. graminearum*, the total starch content was negatively affected by the infection in Cadenza SBEIIa, but not in Sumai3, suggesting that *F. graminearum* is able to attach either HAS or wild-type starch. This is the first research study reporting the interaction between FHB and HAS as a carbon source; thus, more research is needed to replicate and validate such data. Notably, the application of NPF up-regulated some genes involved in the starch biosynthetic pathway in Sumai3 and Cadenza SBEIIa, suggesting that the NPF could act as an elicitor of starch production.

## 4. Materials and Methods

### 4.1. Fungal, Chemical, and Plant Materials

The highly virulent isolates of *F. graminearum* WT3824 and *F. culmorum* UK99 were the reference strains used in this study as causal agents of FHB and FCR, respectively. CH hydrochloride (Merck, Germany; 99% purity, molecular weight 60 kDa, degree of deacetylation 80–90%; CAS number 70694-72-3) and GA (Merck, Germany; >97% purity; CAS number 149-91-7) were used as active antimicrobials; tebuconazole (Merck, Germany; CAS number 107534-96-3) was used as reference fungicide. Cellulose and starch were extracted from the bran and flour of the bread wheat genotype Cadenza SBEIIa, characterized by a high content of HAS instead of conventional starch [14]. Cadenza SBEIIa, its related wild-type genotype Cadenza and Sumai3 (FHB resistant genotypes) were employed for in vivo assays.

### 4.2. Cellulose Extraction and Synthesis of CNC from Bread Wheat Bran

Wheat bran was obtained from the bread wheat cultivar Cadenza SBEIIa and cellulose was extracted by optimizing different protocols already present in the literature [65,66,67]. Five hundred g of wheat bran was accurately rinsed in deionized water until the washing water was clear and dried at 60 °C for 24 h. After that, the biomass was finely ground and sieved with a 40-mesh sieve. The powder was treated in a 70% ethanol solution (1:15 *w*/*v*) for 24 h under continuous stirring. The obtained biomass was rinsed several times with deionized water to remove ethanol traces and dried at 60 °C for 24 h. The biomass was further treated in a 5% NaOH solution (1:20 *w*/*v*) at 70 °C for 4 h under continuous stirring, rinsed again with deionized water to remove any NaOH traces and dried at 60 °C for 24 h. The biomass was further bleached in a 7% NaClO_2_ solution (pH = 4 by adding glacial acetic acid) (1:50 *w*/*v*) at 70 °C for 2 h under continuous stirring. The bleached biomass was accurately rinsed again, dried at 60 °C for 24 h, and subjected to another NaOH treatment (10%) (1:20 *w*/*v*) at 70 °C for 4 h under continuous stirring. The biomass was finally rinsed several times with deionized water, dried at 60 °C for 24 h, and subjected to hydrolysis in a 64% sulfuric acid solution at 45 °C for 90 min under continuous stirring. The hydrolysis was stopped by pouring the hydrolysis solution into a 10 times greater volume of water and it was left to resuspend for 24 h. The suspension was then recovered and put in dialysis tubes until the pH reached 7. After that, CNC aqueous suspension went through an ultrasonic treatment (700 W for 8 min) and was stored at 4 °C after adding a few drops of pure acetone. Cellulose extraction and CNC synthesis were performed in triplicate to statistically validate the protocol’s reproducibility. 

### 4.3. CNC Characterization

CNC dry content was calculated by using the method reported as UNI EN ISO 638:2009. Briefly, 5 mL of backers was weighed before (container weight) and after (initial weight) adding 3 mL of CNC suspension. The suspension was dried at 60 °C for 24 h and the dry weight (final weight) was annotated. The CNC dry content was calculated as:

$$\mathrm{dry}\;\mathrm{content}=\left(\frac{\mathrm{final}\;\mathrm{weight}-\mathrm{container}\;\mathrm{weight}}{\mathrm{initial}\;\mathrm{weight}-\mathrm{container}\;\mathrm{weight}}\right)\times 100$$

The dry content was calculated from three independent cellulose extraction and CNC synthesis procedures, each one consisting of five technical replicates. 

The Fourier Transform Infrared spectra with attenuated total reflection (internal reflection accessory, ATR-FTIR) were recorded using an IRSpirit Infrared Spectrophotometer (Shimadzu Italy, Milan, Italy in the spectral range of 400–5000 cm^−1^ and with a resolution of 4 cm^−1^ at 50 scans. A small quantity of the powdered samples was deposited directly on the diamond ATR crystal plate with no sample processing. Background spectrum was acquired on the empty crystal plate.

UV/VIS absorption spectra were recorded with an Agilent 8453 UV/VIS spectrometer (Agilent Technologies, Santa Clara, CA, United States. Scans were performed over a wavelength range from 200 to 800 nm. Standard cuvettes with optical path length of 1 cm were used and all measurements were performed in triplicate.

X-ray powder diffraction (XRD) data collection was performed with a Bruker D8 Advance diffractometer in Bragg–Brentano geometry, equipped with a Lynxeye XE-T fast detector, using the Cu-Kα (40 kV, 40 mA). Data were collected in the 4–80° 2θ range using a 0.017° step scan and 200 s counting time. The patterns were decomposed in the contributions of cellulose Ib and cellulose II reflections, and the amorphous content by means of a profile fit procedure with pseudo-Voigt functions, in the 5–32° 2θ range, using the Philips ProFit software [68]. Cellulose Ib and cellulose II peak positions were first set up and refined from literature data [69,70]. The relative contributions, in weight percentage (wt%), were assumed proportional to the total integrated intensities per each phase, while the crystallinity index was estimated as the wt% of the crystalline phases.

The morphology of the samples was investigated with a field-emission microscope Zeiss LEO 1525 equipped with a GEMINI column, (Zeiss Microscopy, Oberkochen, Germany). Samples for SEM analysis were prepared by depositing the powder onto an aluminum specimen stub covered with a double-sided adhesive disc. Then, the samples were coated with a chromium layer (8 nm thickness) before imaging (Quorum Q150T ES East Grinstead, West Sussex, UK).

Particle size analysis of CNC was determined using a single particle optical sensing (SPOS) particle sizer Accusizer C770 equipped with an auto dilution system. Samples were prepared by direct dispersion in n-hexane.

### 4.4. Synthesis of Aminated CNC (dCNC-NH_2_)

CNC extracted from wheat bran were then chemically functionalized with the insertion of amino groups on their surface by modifying a known synthetic procedure [71]. Briefly, CNC (5%, *w*/*w*) were dissolved in a binary mixture of ionic liquids, 1-ethyl-3-methylimidazolium acetate ([EMIM][OAc]) and 3-butyl-1-(4-sulfobutyl)-imidazolium hydrogen sulfate ([BSBIM][HSO_4_]) (98:2 mol%) [72] at 60 °C and reacted with glycidol (CNC/glycidol 1:5, *w*/*w*) for 18 h. After dilution and filtration with ethanol, epoxidized CNC were suspended in a 30% aqueous solution of ammonia (5%, *w*/*v*) and allowed to stir for 6 h at 60 °C. The reaction mixture was cooled down and then dialyzed against deionized water for 48 h. The resulting suspension was freeze-dried obtaining dCNC-NH2 as dry powder.

### 4.5. Synthesis and Characterization of the NPF

Starch employed for NPF synthesis was extracted and characterized from Cadenza SBEIIa in a previous work [14].

The antimicrobial NPF formed with CH and GA as active principles, and CNC as carriers, and HAS as excipient were synthesized as follows. The aqueous suspension of co-formulated materials was nebulized and dried using a Buchi mini spray-dryer B-290 (Büchi, Flawil, Switzerland). A suspension of dCNC-NH_2_ with a concentration of 25 mg/mL in deionized water was concentrated under vacuum until a jelly consistency was obtained. Then, a solution of GA in MeOH (0.1 mg/μL) was added over to obtain a 15% *w*/*w* of theoretical loading. The mixture was stirred at room temperature, under dark conditions for 1 h. Then, the solution was kept at 4 °C for 16 h. After ultracentrifugation at 70,000 rpm for 30 min, the supernatant was analyzed by UV/VIS to quantify the amount of GA in solution. The working curve was built in water solution in the following conditions: 2.8 to 21 µg/mL concentration range, λmax = 265 nm (r^2^ = 0.998). After that, a suspension of dCNC-NH_2_/GA in water (3 mg/mL) and Tween^®^ 80 (0.25%, *w*/*v*) was mixed with a suspension in water of CH hydrochloride with the same concentration (3 mg/mL) by using a peristaltic pump with a flow rate of 10 mL/min and homogenized with an Ultra-Turrax homogenizer system. HAS was added to the resulting suspension with a ratio of 1:1 (*w*/*w*). To produce powdered particles, the final suspension was then passed to the spray-dryer apparatus and dried by using the following conditions: inlet temperature: 160 °C; aspirator rate: 50%; feeding pump rate: 1.8 mL/min; air pressure: 35 mm. To quantify the amount of GA in the formulation powder, the following procedure was used. A certain amount of powder was suspended in a mixture of H_2_O/MeOH/HCl 3N (ratio of 1:2:2), ultrasonicated, and stirred at room temperature for 3 h and then passed to UV/VIS analysis as above reported. For the quantification of CH, the formulation was suspended in a mixture of water and acetic acid (5%, *v*/*v*). After filtration, 1 mL of the solution was mixed with 0.1 mL of 0.5 M NaNO_2_ aqueous solution and heated up to 80 °C for 45 min. Then, 1 mL of a dinitrosalicylic acid reagent (DNSA) was added and the resulting yellow solution was allowed to react for further 15 min at 75 °C. Then, the solution was analyzed by using UV/VIS spectrophotometry. The tubes were cooled under tap water and absorbance was measured at 540 nm [73].

The release of GA and CH from the formulation was performed in water at room temperature. For GA, an established amount of formulation was suspended in 3 mL of water with a concentration of 12 mg/mL and transferred to a dialysis tube with an MW cut-off of 14 kDa. The experiment was carried out in 47 mL of water in a 50 mL Erlenmeyer flask, and an aliquot of 3 mL was collected at established times and immediately replaced with the same amount of fresh solvent. The amount of released GA was measured by UV/VIS spectroscopy with the method reported above. For the release of CH, the following procedure was applied. In a falcon vial tube, an established amount of formulation was suspended in 10 mL of water. After centrifugation at 4000 rpm for 10 min (20 °C), the whole supernatant was collected at established times and then replaced with same amount of fresh solvent. After derivatization with DNSA by using the procedure described above, the amount of released CH was measured by UV/VIS spectroscopy.

### 4.6. In Vitro Antifungal Assays

Four different in vitro assays were performed in order to test the inhibitory effect of the NPF against *F. graminearum* and *F. culmorum*. The NPF was tested at 2% (*w*/*v*) in order to have CH at 0.5% (*w*/*v*) and GA at 0.05% (*w*/*v*) in the final solution, which were the optimal concentrations to show antifungal activities in preliminary experiments. Moreover, tebuconazole was tested as reference fungicide at the suggested filed dose from commercial formulations (0.06% *w*/*v*).

#### 4.6.1. The 96-Microtiter-Plates Assay

Fresh mycelium of *F. graminearum* and *F. culmorum* grown on Potato Dextrose Agar (PDA) was transferred to Synthetic Nutrient Poor Agar (SNA) and cultured at 21 °C to obtain macroconidia [74]. After 10 days on SNA, the conidia were scraped with a glass rod after pipetting 1 mL of sterile distilled water onto the surface of a Petri dish. The conidial suspension was recovered, and the concentration was measured by using a Thoma Chamber (0.100 mm depth and 0.0025 mm^2^). The NPF and tebuconazole were dissolved in sterilized Potato Dextrose Broth (PDB) at the previously cited concentrations, the pH values were controlled at around 5, and were filtered by using a sterile syringe filter with a pore size of 0.2 µm. The obtained solutions were pipetted into the microtiter plates and the conidial suspension was added to each well in order to obtain a final concentration of 1 × 10^5^ conidia/mL. The plates were incubated at 21 °C in the dark for 48 h. The absorbance from the fungal biomass was measured at OD_450_ by using a DR-200B Microplate reader (Diatek instruments, Wuxi City, China). Mock (untreated conidial suspension cultured on PDB) and blank (PDB supplemented with the NPF, PDB supplemented with tebuconazole, and only PDB) controls were also included. Data were obtained from three independent experiments, each one consisting of four replicates. 

#### 4.6.2. Incorporated Medium Assay

Sterile PDA was supplemented with the NPF or tebuconazole at the previously cited concentrations. The PDA incorporated with the compounds was poured on Petri dishes and allowed to solidify. Afterward, a 5 mm plug from a fresh culture of *F. graminearum* or *F. culmorum* was placed at the center of the Petri dish and cultured in the dark at 21 °C for 7 days. Mock control was also included. After 7 days, the mycelial growth was evaluated by measuring four fungal colony diameters from each plate. Data were obtained from three independent experiments, each one consisting of four replicates for each substance and concentration tested.

#### 4.6.3. Cellular Aggregation Assay

The aggregation assay was performed to evaluate the ability of the NPF to inhibit the cell-to-cell adhesion. The assay was performed using a standard report already published [75]. *F. graminearum* and *F. culmorum* macroconidia were obtained as described in Section 4.6.1. The NPF and tebuconazole were dissolved in sterilized distilled water at the previously cited concentrations and filtered by using a sterile syringe filter with a pore size of 0.2 µm. The obtained solutions were pipetted into the microtiter plates and the conidial suspension was added to each well in order to obtain a final concentration of 1 × 10^6^ conidia/mL. The plates were incubated at 21 °C in the dark, and the absorbance from the fungal biomass was measured at 0 h, 24 h, 48 h, and 72 h post incubation at OD_620_ by using a DR-200B Microplate reader (Diatek instruments). Mock (untreated conidial suspension in sterile distilled water) and blank (sterile distilled water supplemented with the NPF, sterile distilled water supplemented with tebuconazole, and only sterile distilled water) controls were also included. Higher absorbance values compared to the mock control were associated with more aggregated conidia. Data were obtained from three independent experiments, each one consisting of four replicates.

#### 4.6.4. Cellular Adhesion Assay

The adhesion assay was performed to evaluate the ability of the NPF to potentially inhibit conidial adhesion to the host tissues. The assay was performed following reports already published with some modifications [76]. *F. graminearum* and *F. culmorum* macroconidia were obtained as described in Section 4.6.1. The NPF and tebuconazole were dissolved in sterilized distilled water at the previously cited concentrations and filtered by using a sterile syringe filter with a pore size of 0.2 µm. The obtained solutions were pipetted into the microtiter plates and dried at 60 °C for 24 h in order to favor the adhesion of the active compounds (NPF and tebuconazole) to the plastic surface. After that, 200 µL of 1 × 10^6^ conidia/mL suspension were pipetted into each well and incubated at 21 °C for 3 h. At the end of the incubation time, the conidial suspension was removed with a pipette and 200 µL of sterile distilled water was added to each well. The microtiter plates were bath-sonicated for 15 min to release the conidia cells attached to the plastic surface. The 1:100 dilution from the sonicated suspension was pipetted into new microtiter plates containing 200 µL of PDB and incubated at 21 °C for 24 h. The absorbance from the fungal biomass was measured at OD_450_ by using a DR-200B Microplate reader (Diatek instruments). Mock (untreated conidial suspension cultured on PDB) and blank (PDB) controls were also included. Higher absorbance values compared to the mock control were associated with more adhered conidia. Data were obtained from three independent experiments, each one consisting of four replicates.

### 4.7. In Vivo Biocompatibility and Antifungal Assays

Biocompatibility of the NPF was assessed by coating the kernels of Sumai3, Cadenza, and Cadenza SBEIIa and by evaluating the % of germination, Nitrogen Balance Index (NBI), and dry biomass. The in vivo antifungal ability of the NPF was assessed by artificially inoculating *F. graminearum* and *F. culmorum* on plants. The impact on yield was assessed by weighting inoculated and treated harvested spikes.

#### 4.7.1. In Vivo Biocompatibility

Plants were grown in a climatic chamber under controlled conditions. The surface of the kernels of the bread wheat genotypes Sumai3, Cadenza, and Cadenza *SBEIIa* were sterilized with sodium hypochlorite (0.5% *v*/*v*) for 20 min and then rinsed twice for 5 min in sterile distilled water. After that, the kernels were soaked for 1 h in the solution containing the NPF at 2% and in distilled water as mock control under constant stirring and allowed to dry for 1 h. Then, the kernels were transferred to 40 × 20 cm pots, filled with TYPical Brill soil, and grown at 18/12 °C (day/night) with 12–14 h of light for 14 days. At the end of the experiment, the germinated kernels were counted, and a kernel was considered germinated when the coleoptile was visible. Nitrogen Balance Index (NBI) (DUALEX Scientific+™) relating to the chlorophyll and flavonoid ratio was measured by positioning the instrument in the center of the leaf and the meter was shielded from direct sunlight. Afterward, the seedlings were removed from the soil, gently washed with distilled water, dried at 60 °C for 24 h, and weighed to evaluate the dry biomass. Data were obtained from three independent experiments organized as a complete randomized block, each one consisting of 20 plants for each experimental group (wheat genotype × treatment). 

#### 4.7.2. In Vivo Antifungal Assay

The artificial inoculation trials were conducted in a climatic chamber under controlled conditions.

For FHB experiments, seeds of the bread wheat genotypes Sumai3, Cadenza, and Cadenza SBEIIa were germinated into pots (17 cm diameter, 20 cm height) filled with TYPical Brill soil grown at 18/12 °C (day/night) from tillering to heading with 12–14 h of light. During flowering time, the conditions were set at 21 °C, 55% relative humidity during daytime and 17 °C, 55% relative humidity during the night with a 16 h photoperiod. The NPF at 2% and tebuconazole at 0.06% were prepared in distilled water. *F. graminearum* macroconidia were obtained as described in Section 4.6.1. The antifungal compounds were sprayed on spikes (wetted until runoff) 48 h before inoculation. Plants subjected only to artificial inoculation and mock treatment were sprayed with distilled water. Plants were subjected to artificial inoculation at anthesis (Zadok stage 69) [77] by homogenously spraying a conidial suspension of 1 × 10^5^ conidia/mL supplemented with 0.05% Tween 20, while mock plants were sprayed with a solution of 0.05% Tween 20 (wetted until runoff). The disease incidence and severity of the FHB (%) were determined by counting the number of diseased spikes and the number of bleached spikelets in diseased spikes as well as the total number of spikes and spikelets, respectively, from 3 to 21 dpi. Data were obtained from three independent experiments organized as a complete randomized block, each one consisting of 20 plants for each experimental group (wheat genotype × treatment).

For FCR experiments, seedlings of the bread wheat genotypes Sumai3, Cadenza, and Cadenza SBEIIa were grown in jiffy pots filled with TYPical Brill soil and were arranged in plastic trays covered with a plastic film to maintain high relative humidity, at 21 °C with a 12–14 h of light. During the first-leaf stage, the conditions were set at 21 °C, 55% relative humidity during daytime and 17 °C, 55% relative humidity during the night with a 16 h photoperiod. The NPF at 2% and tebuconazole at 0.06% were prepared in distilled water. *F. culmorum* macroconidia were obtained as described in Section 4.6.1. The antifungal compounds were sprayed on the stem base leaf sheath (wetted until runoff) 48 h before inoculation. Plants subjected only to artificial inoculation and mock treatment were sprayed with distilled water. Plants were subjected to artificial inoculation at anthesis (Zadok stage 11) [77] by homogenously pipetting on the coleoptile 20 µL of a conidial suspension of 1 × 10^5^ conidia/mL supplemented with 0.05%, while 20 µL of a solution of 0.05% Tween 20 was pipetted on mock plants. Disease symptoms were assessed at 5, 8, 12, and 20 days post-inoculation, using two parameters, symptom extension (SE, in millimeters) and browning index (BI) of the infected tissues (visually rating as 0 for no symptoms, 1 for bleaching, 2 for slight necrosis, 3 for severe necrosis, and 4 for tissue disruption and visible mycelium). The Disease Index (DI) was then calculated as follows:

DI=SE×BI

Data were obtained from three independent experiments organized as a complete randomized block, each one consisting of 20 plants for each experimental group (wheat genotype × treatment).

### 4.8. Evaluation of Resistance Induction by Real-Time qPCR

The relative expression level of genes listed in Table S4 was evaluated as involved in the resistance induction and starch biosynthesis. Treated, inoculated, and mock spikes were harvested 48 h after the inoculation and immediately stored in liquid nitrogen and at −80 °C. Then, 100 mg of fine powder was subjected to RNA extraction following the instructions provided by GRS Total RNA Kit-Plant (GRiSP Research Solutions, Portugal). The RNA was resuspended in RNase-free sterile distilled water and immediately poured onto ice and quantified with Qubit™ RNA BR Assay Kit (Thermo Fisher Scientific, Waltham, United States. The synthesis of cDNA was performed using 500 ng of RNA following the instructions provided by Xpert cDNA Synthesis Supermix with a gDNA eraser (GRiSP Research Solutions, Porto, Portugal) in a final volume of 20 µL. To ensure that the synthesis of the cDNA and the elimination of the gDNA had succeeded, a reverse transcription PCR (RT-PCR) of *T. aestivum* Actin (*TaACT*) (containing an intron in the amplified sequence) was performed following the instructions provided by GoTaqR Green Master Mix (Promega, Madison, United States) in a total volume of 10 µL. The amplification conditions consisted of (i) an initial denaturation step of 2 min at 95 °C; (ii) 35 cycles of 30 s denaturation at 95 °C; (iii) 40 s of annealing at 60 °C; (iv) 30 s of elongation at 72 °C; and (v) a final elongation step of 5 min at 72 °C. The amplification run included a no-template control (NTC) and a genomic DNA (gDNA) control. The amplicons were visualized on 1.5% agarose gel. Table S4 shows the list of target genes, accession numbers, their functions, the corresponding primer pairs, and their references used to perform the RT-qPCR. For the primer pairs designed in this study, sequence-similarity searches were carried out starting from known sequences obtained from Gramene (www.gramene.org, accessed on 18 October 2021). The sequences were submitted to BLASTn (https://blast.ncbi.nlm.nih.gov/) analysis in order to extract the corresponding cDNA. Primers for Real-Time qPCR were designed inside the exonic regions by using Primer-BLAST (https://blast.ncbi.nlm.nih.gov/tools/primer-blast/). The optimal annealing temperature was assessed by running a gradient PCR for each primer pair.

The relative expression levels of the target genes were calculated on the basis of the Cq values of the four technical replicates derived from three independent biological replicates for each wheat genotype and compound by applying the equation:

$$Relative\;expression\;=\;2^{-\Delta \Delta Cq},$$

using *TaFNR*, *TaTUB*, and *TaACT* as the reference genes and the mock treatment to normalize the relative expression levels [78]. The RT-qPCR was performed following the instructions provided by Rotor-Gene Q (Qiagen, Hilden, Germany) and Xpert Fast SYBR (uni) MasterMix (GRiSP Research Solutions, Porto, Portugal), in a final volume of 10 µL. The amplification conditions consisted of (i) an initial denaturation step of 3 min at 95 °C; (ii) 40 cycles of 5 s denaturation at 95 °C; (iii) 30 s of annealing at 60 °C; and (iv) 20 s of elongation at 72 °C. A final melt cycle (70–99 °C) was performed to confirm the unicity of the amplicons. NTC controls were included and the amplification was considered negative when a value of Cq ≥ 38 was detected [79].

### 4.9. Quantification of Fungal Biomass and Impact on Yield

For yield evaluation, thirty spikes from each experimental group (wheat genotype × treatment) (10 spikes from each independent replicate) were harvested and weighed (g). For quantification of fungal biomass by Real-Time qPCR, ten spikes and plantlets from each independent replicate of each experimental group (wheat genotype × treatment) were homogenously milled in liquid nitrogen into a fine powder. Then, 100 mg of *F. graminearum* and *F. culmorum* fresh mycelium and 100 mg of plant material from Sumai3, Cadenza, and Cadenza SBEIIa were subjected to total DNA extraction following the protocol for the GRS Genomic DNA Kit-Plants (GRiSP Research Solutions, Porto, Portugal). DNA was quantified with a Qubit™ fluorometer 1.01 (Invitrogen, Waltham, United States) using the Qubit™ dsDNA BR Assay Kit (Thermo Fisher Scientific, Waltham, United States). DNA from inoculated samples was diluted to 10 ng/μL, while fungal and wheat calibration curves were obtained by preparing four serial 1:10 dilutions (1:1, 1:10, 1:100, 1:1000) from fresh fungal mycelium and uninoculated wheat material DNAs. RT-qPCR was performed following the instructions from Rotor Gene Q (Qiagen, Hilden, Germany) and Xpert Fast SYBR (uni) Master Mix (GRiSP Research Solutions, Porto, Portugal). RT-qPCR amplification conditions included an initial denaturation step of 3 min at 95 °C; 40 cycles of 5 s denaturation at 95 °C, 30 s of annealing at 60 °C; and 20 s of elongation at 72 °C. A final melt cycle (70–99 °C) was performed to confirm the amplicon’s unicity. All the primer pairs used are listed in Table S5. Data were obtained from four technical replicates derived from three biological replicates. Results were expressed as ng of fungal DNA per ng of plant DNA [80,81].

### 4.10. Quantification of Total Starch from Mature Treated and Infected Kernels

Total starch was determined from whole flour obtained from the wheat spike, using the Total Starch Assay kits (AA/AMG) (Megazyme, Irishtown, Ireland) and following the protocol specified for “samples containing also resistant starch”. For each analysis, three biological samples consisting of a bulk of ten spikes were measured.

### 4.11. Statistical Analysis

Data were subjected to one-way analysis of variance (ANOVA). One level of significance (*p* < 0.01) was computed to assess the significance of the F values. A pairwise analysis was carried out using the Tukey Honestly Significant Difference test (Tukey test) at the 0.99 confidence level. Statistical analyses were performed using SYSTAT12 software (Systat Software Inc., San Jose, CA, USA). The heatmaps were generated to represent the relative expression levels of wheat genes after the plant treatments, while the principal component analysis (PCA) was carried out to evaluate the genotype × compound effects on controlling FHB and FCR by plotting FHB incidence and severity at 21 dpi, the spikes’ weight, and the ng of fungal DNA/ng of plant DNA or by plotting FCR incidence and severity at 20 dpi and the ng of fungal DNA/ng of plant DNA. PCA and heatmap were carried out using ClustVis software (Tartu, Estonia) [82].

## 5. Conclusions

The present research work investigated the employment of an NPF formed with CNC and HAS extracted from wheat and serving as a nano-carrier and excipient for delivering two active compounds, CH and GA, for the sustainable management of FHB and FCR. The study demonstrated that the circular economy and nanotechnology are two powerful weapons to reach sustainability in plant disease management, since we valorized wheat wastes (bran) to obtain a useful carrier and excipient for the efficient delivery of green pesticides. For such reasons, despite nano-agrochemicals still being in their infancy, probably because of their production cost and lack of awareness among farmers, this technology will one day lead the market due to investment and innovation in more economically sustainable production. Indeed, several worldwide companies (for example Borregaard, Nanografi Nano technology, Cellulose Lab, Celluforce, Sappi) are investing in the CNC market for several applications with the aim of developing affordable production systems.

In the present study, the NPF showed two antifungal modes of action: it directly provoked toxicity on fungal conidia and mycelium, but also mechanically interacted with conidia to reduce their fitness on a putative plant surface. During in vivo experiments, the NPF efficiently decreased FHB symptoms and the accumulation of fungal biomass, and drastically boosted innate immunity in the bread wheat genotype Cadenza SBEIIa. The application of the NPF against FCR reduced symptoms development, even though the obtained data were not clearly unambiguous. Despite this, the employment of Cadenza SBEIIa may be particularly interesting to control pathogen spread into plantlets. These findings could open novel research frontiers in plant breeding to understand the presence of interesting alleles conferring FHB or FCR resistance or tolerance, as Cadenza SBEIIa was particularly responsive to plant defense elicitors and efficient in counteracting *F. culmorum* biomass spread. 

## Data Availability

All the relevant data are presented in this work.

## Supplementary Materials

The following supporting information can be downloaded at https://www.mdpi.com/article/10.3390/plants12061223/s1, Table S1. Dry content (%) and yield (%) of CNC extracted from the bran of the wheat bread genotype Cadenza. Data are expressed as mean ± standard deviation (SD) that were calculated from three independent extraction protocols. Table S2. GA loading on CNC-NH2. GA binding was indirectly calculated by UV/VIS quantification of the supernatant after ultracentrifugation as the difference between the theoretical loading (15% *w*/*w*) and the amount of GA in solution. The GA loading efficiency was calculated as percentage of the bound GA amount and the GA amount added. Table S3. Characterization of the nanostructured particle formulation (NPF). Batch size accounts for the CNC-NH2/GA, CH, HSA (1:1:2, *w*/*w*/*w*) and Tween 80 (T80, 0.25% *w*/*w*) amount fed to the spray dryer. Yield was calculated as the ratio between the amount of the powder collected and the starting batch size. GA and CH contents represent the bioactive compound amount per unit of weight of formulation. GA and CH recovery was calculated as the ratio between the amount of the bioactive compounds loaded and that recovered after spray-drying. Table S4. List of *Triticum aestivum* selected genes, accession numbers, gene function, primer pairs, amplicon length from DNA and cDNA and primer pairs references [19,21,78,81,83,84,85,86,87,88,89,90,91,92,93,94]. Table S5. List of *Fusarium graminearum*, *Fusarium culmorum* and *Triticum aestivum* selected genes, accession numbers, gene function, primer pairs, amplicon length from DNA and primer pairs references used for fungal biomass quantification [80,94,95]. Supplementary Figure S1. *In planta* biocompatibility assay. Boxplots of the germination assay (a); the % of germinated kernels was recorded at 14 days after germination. Boxplots of the NBI measurements (b); NBI values were recorded at 14 days after germination. Boxplots of the dry biomass measurements (c); plantlets were collected at 14 days after germination and weighted after drying at 60 °C for 24 h. The boxplots have been obtained by plotting the data collected from three independent experiments, each one consisting of twenty plantlets for each experimental group. Different letters (a, b) into the subfigures refer to the statistical analysis performed using one-way analysis of variance (ANOVA) with the Tukey test at a confidence level of 0.99 and *p* < 0.01, while “n. s.” stands for “not significant”. Supplementary Figure S2. Representative pictures of treated and inoculated spikes with Fusarium graminearum at 21 days post inoculation (dpi) (a) and of treated and inoculated coleoptile with Fusarium culmorum at 20 dpi. Supplementary Figure S3. 1.5% agarose gel of selected genes amplified by using the primer pair listed in Table S4. (M) DM2300 ExcelBand™ 100 bp+3K DNA Ladder (Smobio); (1) *TaICS*, (2) *TaCHI*, (3) *TaLOX*, (4) *TaWRKY*, (5) *TaPR5*, (6) *TaNPR1*, (7) *TaPDF*, (8) *TaPR10*, (9) *TaPR12*, (10) *TaPR14*, (11) *TaPEN1*, (12) *TaFMO1*, (13) *TaLLP1*, (14) *TaCAMTA3*, (15) Negative control (a). 1.5% agarose gel of RT-PCR of *TaACT* amplified from Sumai3, Cadenza and Cadenza SBEIIa. (M) DM2300 ExcelBand™ 100 bp+3K DNA Ladder (Smobio); (1) and (2) *TaACT* from Sumai3, (3) and (4) *TaACT* from Cadenza, (5) and (6) *TaACT* from Cadenza SBEIIa, (7) Negative control, (8) *TaACT* from gDNA (b). Supplementary Figure S4. Standard curves for the TaACT in Sumai3 (a), Cadenza (b) and Cadenza SBEIIa (c) and Tri5 (d) genes for the Real-Time qPCR quantification of the fungal biomass. The standard curve for the Tri6 quantification was already obtained and validated in a previous work [22]. Supplementary Figure S5. Principal Components Analysis (PCA) carried out to investigate the effective contribution on controlling FHB and FCR. The PCA on FHB data was carried out by plotting FHB incidence and severity at 21 dpi, impact on final yield and ng of fungal DNA/ng of plant DNA (a). The PCA on FCR data was carried out by plotting FCR incidence and severity at 20 dpi and ng of fungal DNA/ng of plant DNA (b). PCA were conducted by using ClustVis software (Tartu, Estonia).
