# Supplementary material for: Boosting Immunity and Management against Wheat Fusarium Diseases by a Sustainable, Circular Nanostructured Delivery Platform
Source: Plants (Basel). 2023 Mar 8;12(6):1223. doi: 10.3390/plants12061223 (PMC10054448; doi:10.3390/plants12061223)
Supplement: Supplementary file 1 [file plants-12-01223-s001.zip › plants-2239456-supplementary.pdf]

Supplementary Materials - Tables

Table S1. Dry content (%) and yield (%) of CNC extracted from the bran of the wheat bread genotype Cadenza. Data are expressed as mean ± standard deviation (SD) that were calculated from three independent extraction protocols.

	I replicate	II replicate	III replicate	Average ± standard deviation
**Dry content (%)**	0.4	0.8	0.3	0.5±0.3
**Yield (%)**	26.6	10.1	9.3	15.3±9.8

Table S2. GA loading on CNC-NH2. GA binding was indirectly calculated by UV/VIS quantification of the supernatant after ultracentrifugation as the difference between the theoretical loading (15% w/w) and the amount of GA in solution. The GA loading efficiency was calculated as percentage of the bound GA amount and the GA amount added.

Entry	$CNC-NH_2$ (g)	Theoretical loading (g)	GA in solution (g)	GA content (% w/w)	Loading efficiency (% w/w)
**1**	0.5	0.088	0.041±	8.6	53
**2**	0.75	0.132	0.070±	7.6	47
**3**	1.5	0.265	0.106±	9.6	60
**4**	3.0	0.530	0.141±	11.5	73

Table S3. Characterization of the nanostructured particle formulation (NPF). Batch size accounts for the CNC-NH2/GA, CH, HSA (1:1:2, w/w/w) and Tween 80 (T80, 0.25% w/w) amount fed to the spray dryer. Yield was calculated as the ratio between the amount of the powder collected and the starting batch size. GA and CH contents represent the bioactive compound amount per unit of weight of formulation. GA and CH recovery was calculated as the ratio between the amount of the bioactive compounds loaded and that recovered after spray-drying.

Entry	Batch size (g)	Dried powder (g)	Yield (%)	GA recovery (% w/w)	GA content (% w/w)	CH recovery (% w/w)	CH content (% w/w)
**1**	2.083 (0.083 T80)	0.952	46	18.6±	1.5±	54.0±	29.5±
**2**	3.125 (0.125 T80)	1.310	42	20.6±	1.8±	44.9±	26.8±
**3**	6.250 (0.250 T80)	2.780	44	22.1±	1.9±	32.7±	18.4±
**4**	12.500 (0.500 T80)	7.92	63	49.2±	2.9±	69.6±	27.5±

Table S4. List of *Triticum aestivum* selected genes, accession numbers, gene function, primer pairs, amplicon length from DNA and cDNA and primer pairs references.

Gene and Accession number	Function	Primer pairs (5′-3′)	bp (DNA)	bp (cDNA)	Reference
TaMAPK AF079318	Mitogen activated protein kinase	CATCGACGTCTGGTCCGT GTCCTCGTTCCGGATGAATC	154	154	[21]
TaICS XM_020328818.2	Isochorismate synthase	TTCAGCTCCACCAAACCAACCA GGTTTGCCCACTGAAGAAGCG	195	99	[83]
TaCHI XM_020330684.2	Endochitinase	GCGTGTACTTCCTGTCGCAGTA CGGCCAACCCCAACTTC	243	155	[84]

TaPR1 AJ007348	Pathogenesis related protein 1	ACTACGACTACGGGTCCAACA TCGTAGTTGCAGGTGATGAAG	145	145	[85]
TaLOX XM_020345935.2	Lipoxigenase	AACAAGTTCGCCGTCACCTT TTGTCGAGGGTGATGGTCTT	115	115	[81]
TaWRKY XM_020332576.2	Transcription factor	CAAGGCAACAAATCGTGTACATG GAGTCCTACTGAACTGAGCTACCG A	120	120	[86]
TaPR5 XM_037606909.1	Thaumatin-like protein	AGGTAATTTTTTATTGCCCTGTAC TG TTACAGCCGCCGTACTACATGT	86	86	[86]
TaAOS AY196004	Allene Oxide Synthase	TCGGGCGTATTGCTGAGG TGCAGCAGCTTGCTTCTCTC	352	352	[21]
TaPR2 Y18212	B-1,3-endoglucanase	AACGTGCGCCCCTACTACC GCGTCGAACAGGCTCGTGTA	398	398	[21]
TaCDPK KU516994	Calcium dependent protein kinase	CTTCTTTGTGGTGTCCCTCC GCTGTCAAACGCCTCCTT	436	436	[21]
TaRBOH AY561153	NADPH oxidase	TTGTTGGATTAGGAATTGGTGCT TGATCCATGTCGGCAATCTC	441	280	[21]
TaPAL X99705	Phenylalanine ammonia lyase	CGATGCTCGTCCGAGTCAAT CATGACCTCACAGAAGACGC	407	407	[19]
TaNPR1 KU736862.1	Non-expresser of PR genes 1	AAAACAGTGGAACTGGGCCG TTATCGGCCGTGTCTTTGCT	200	200	This study
TaPDF KJ551516.1	JA-mediated defensin	ACTACGAGCGCAAGTGCTTCT ATCATGGGACGTGTCTCGGC	89	89	This study
TaPR10 CA684431	Pathogenesis related protein 10 (RNAse)	TTAAACCAGCACGAGAAACATCA G ATCCTCCCTCGATTATTCTCACG	158	158	[87]
TaPR12 AB089942.1	Defensin	GATGCTTGCTACTGGGCTAGA AGCACAAACTAAAGCCACGAA	186	186	[88]

TaPR14 AK449210.1	Lipid transfer protein	ACGTAGGTACTCCTCTCGCTGT GTTGATCGACCACTTCTTCTCG	148	148	[85]
TaPEN1 BJ285699	Syntaxin	ATCTCTTGGAGTGTGTCGAGCAC AAGCTGATAGAAACAGGCGACAG T	167	91	[86]
TaFMO1 KF924400	Flavin-dependent monooxygenase 1	CCGGTATTGCCATGGATGC AATTTTGCAGCATCTGATCGAA	157	157	[89]
TaLLP1 XM_037581219.1	Legume lectin-like protein 1	AGGAGTGCCCCGTCAAGATTG TCGTAGGTGCCGCTGATGAAG	271	271	[90]
TaCAMTA3 XM_037620416.1	Calmodulin-binding transcription activator 3	GGGGAGGAGTGACGACGAC CGCAGCCATCGATTCTGA	530	141	[91]
TaAGPL DQ406820	ADP-glucose-pyrophosphorylase large subunit	GGCTCCAACGTACACCTCAA CTGGTCGCCTCTTTCCATGT	168	78	[92]
TaAGPS AF492644	ADP-glucose-pyrophosphorylase small subunit	CCGGAGATCACCTGTACCGA GTCGCACGTTCCTCATCCAT	109	109	[92]
TaGBSII AF109395	Granule bound starch synthase	CACAGAATGCCAGAGGCATAG GAACAGATGGGAATCACTCCA	245	151	[93]
TaAInv AM295169	Alkaline invertase	CCCCCTTGTGAATGAGGCATGGGA ACCCACAGGTTGGCCACGAAAATG	70	70	[92]
TaACT AB181991	Actin	TCCTGTGTTGCTGACTGAGG GGTCCAAACGAAGGATAGCA	350	236	[94]
TaTUB TAU76745	B-tubulin	CGAGGAGGGCGAGTACGA AGCAAAGCACGACATGGACAT	79	79	[78]
TaFNR AJ457980	Ferredoxin-NADP(H)-oxidoreductase	CACCGGCCCAGTGATCTT AAGGGCGTCTGCTCCAACT	259	69	[78]

Table S5. List of *Fusarium graminearum*, *Fusarium culmorum* and *Triticum aestivum* selected genes, accession numbers, gene function, primer pairs, amplicon length from DNA and primer pairs references used for fungal biomass quantification.

Gene and Accession number	Function	Primer pairs (5′-3′)	bp (DNA)	Reference
TaACT AB181991	Actin	TCCTGTGTTGCTGACTGAGG GGTCCAAACGAAGGATAGCA	350	[94]
FgTRI6 AB017495.1	Trichothecene transcriptional regulator	TCTTTGTGAGCGGACGGGACTTTA ATCTCGCATGTTATCCACCCTGCT	245	[80]
FcTRI5 AY130291	Trichodiene synthase	CACTTTGCTCAGCCTCGCC CGATTGTTTGGAGGGAAGCC	76	[95]

Supplementary Materials – Figures

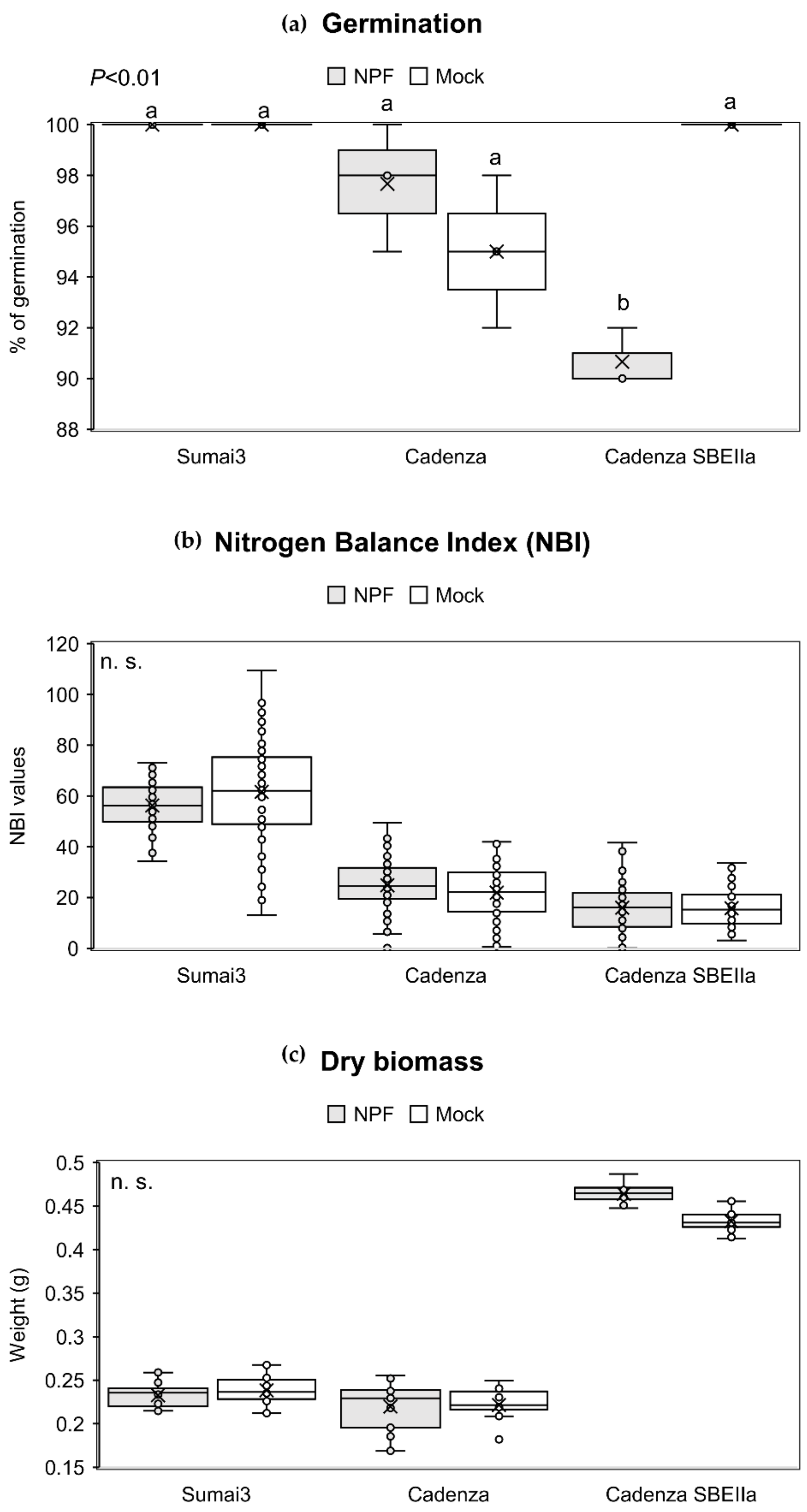

Supplementary Figure S1. *In planta* biocompatibility assay. Boxplots of the germination assay (a); the % of germinated kernels was recorded at 14 days after germination. Boxplots of the NBI measurements (b); NBI values were recorded at 14 days after germination. Boxplots of the dry biomass measurements (c); plantlets were collected at 14 days after germination and weighted after drying at 60°C for 24 h. The boxplots have been obtained by plotting the data collected from three independent experiments, each one consisting of twenty plantlets for each experimental group. Different

letters (a, b) into the subfigures refer to the statistical analysis performed using one-way analysis of variance (ANOVA) with the Tukey test at a confidence level of 0.99 and $P<0.01$, while "n. s." stands for "not significant".

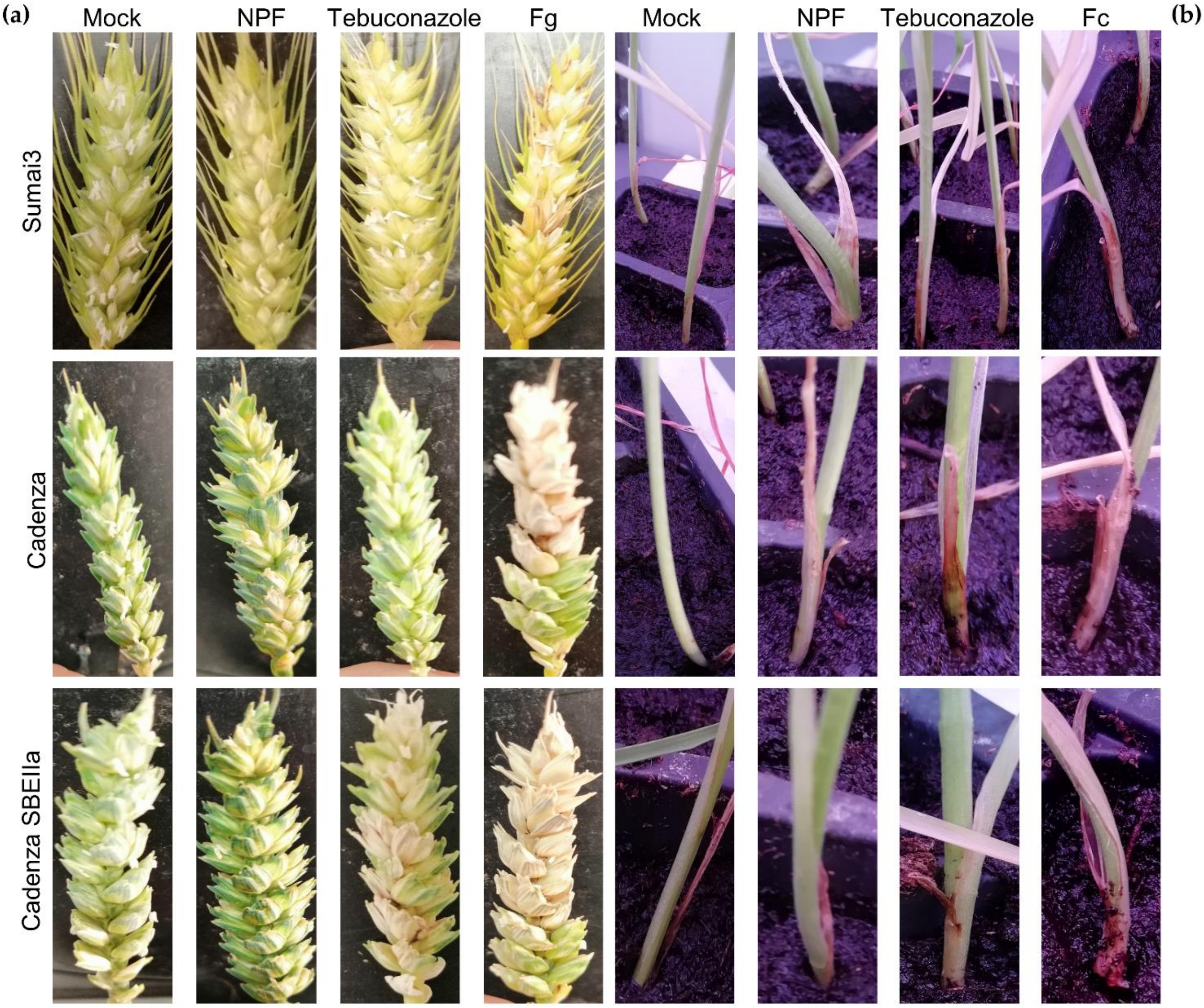

Supplementary Figure S2. Representative pictures of treated and inoculated spikes with *Fusarium graminearum* at 21 days post inoculation (dpi) (**a**) and of treated and inoculated coleoptile with *Fusarium culmorum* at 20 dpi.

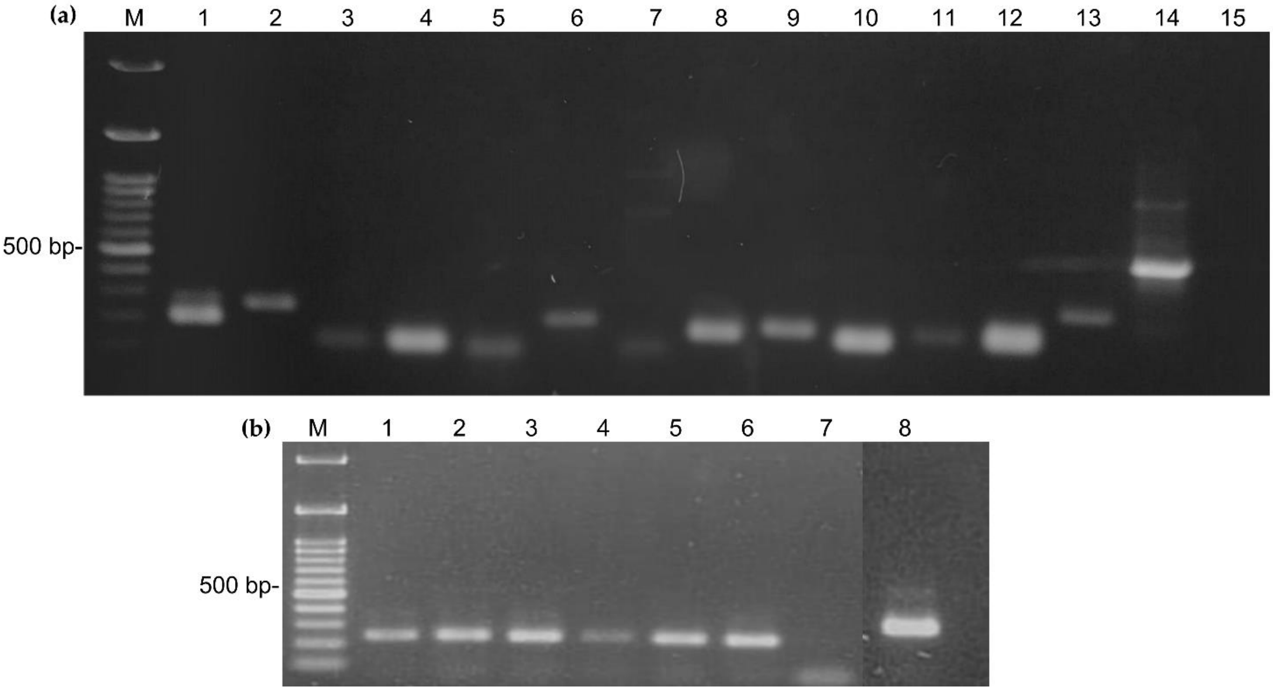

Supplementary Figure S3. 1.5% agarose gel of selected genes amplified by using the primer pair listed in Table S4. M) DM2300 ExcelBand™ 100 bp+3K DNA Ladder (Smobio); 1) *TaICS*, 2) *TaCHI*, 3) *TaLOX*, 4) *TaWRKY*, 5) *TaPR5*, 6) *TaNPR1*, 7) *TaPDF*, 8) *TaPR10*, 9) *TaPR12*, 10) *TaPR14*, 11) *TaPEN1*, 12) *TaFMO1*, 13) *TaLLP1*, 14) *TaCAMTA3*, 15) Negative control (**a**). 1.5% agarose gel of RT-PCR of *TaACT* amplified from Sumai3, Cadenza and Cadenza SBEIIa. M) DM2300 ExcelBand™ 100 bp+3K DNA Ladder (Smobio); 1) and 2) *TaACT* from Sumai3, 3) and 4) *TaACT* from Cadenza, 5) and 6) *TaACT* from Cadenza SBEIIa, 7) Negative control, 8) *TaACT* from gDNA (**b**).

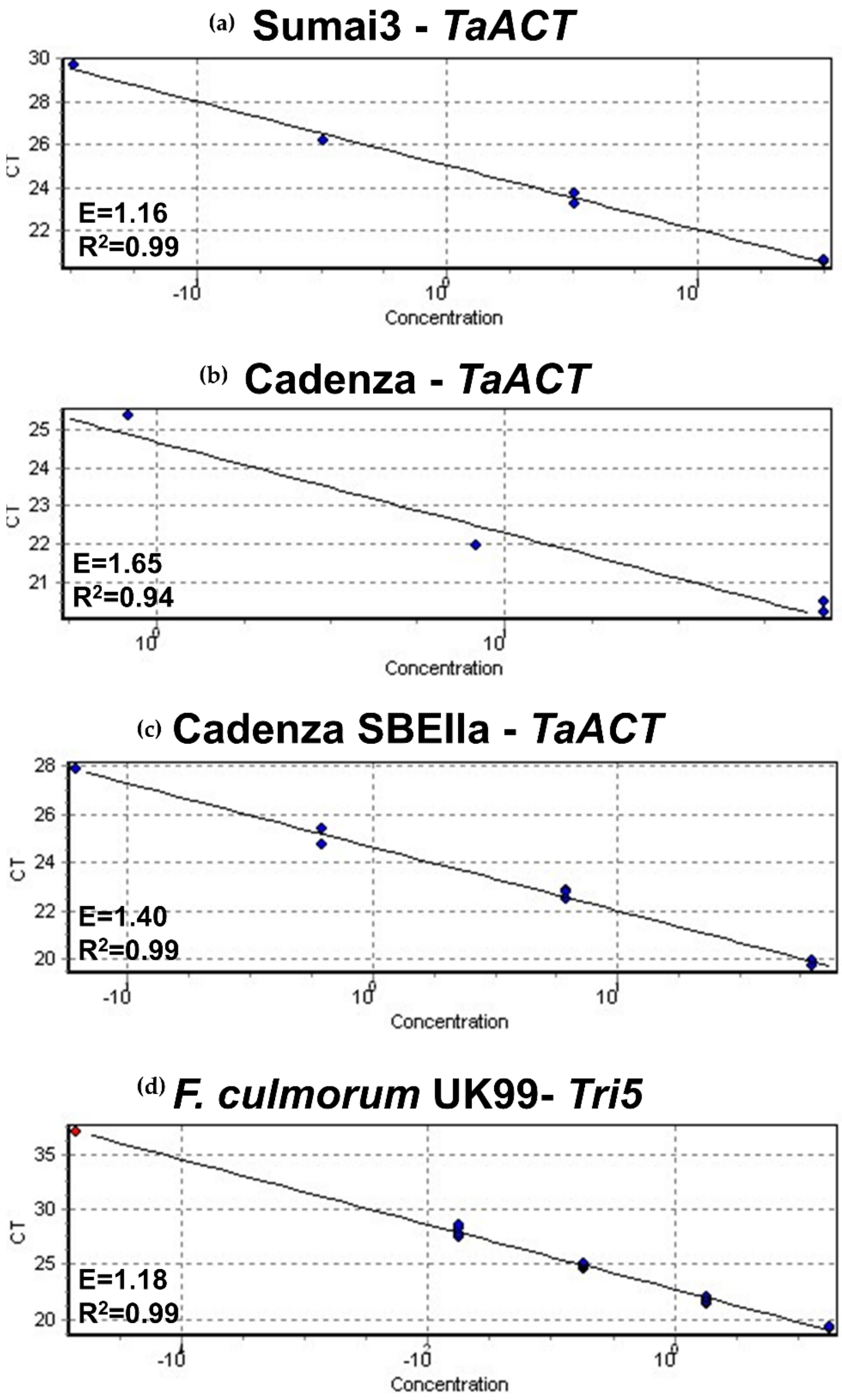

Supplementary Figure S4. Standard curves for the *TaACT* in Sumai3 (a), Cadenza (b) and Cadenza SBEIIa (c) and *Tri5* (d) genes for the Real-Time qPCR quantification of the fungal biomass. The standard curve for the *Tri6* quantification was already obtained and validated in a previous work [22].

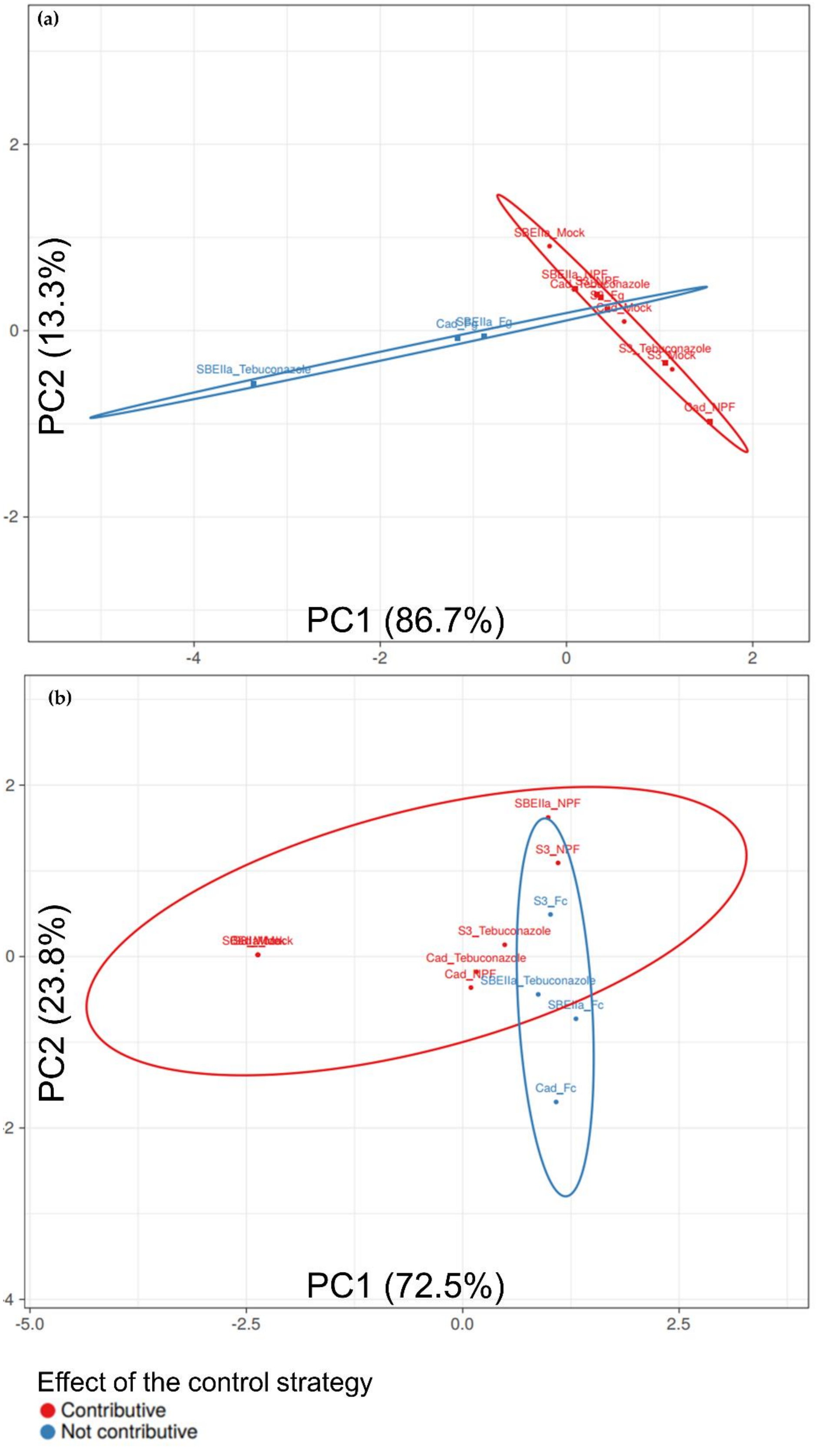

Supplementary Figure S5. Principal Components Analysis (PCA) carried out to investigate the effective contribution on controlling FHB and FCR. The PCA on FHB data was carried out by plotting FHB incidence and severity at 21 dpi, impact on final yield and ng of fungal DNA/ng of plant DNA (a). The PCA on FCR data was carried out by plotting FCR

incidence and severity at 20 dpi and ng of fungal DNA/ng of plant DNA (b). PCA were conducted by using ClustVis software (Tartu, Estonia).

References

21. Francesconi, S.; Balestra, G.M. The modulation of stomatal conductance and photosynthetic parameters is involved in Fusarium head blight resistance in wheat. *PLoS One* **2020**, *15*, e0235482, doi:10.1371/journal.pone.0235482.
83. Kovács, V.; Gondor, O.K.; Szalai, G.; Darkó, É.; Majláth, I.; Janda, T.; Pál, M. Synthesis and role of salicylic acid in wheat varieties with different levels of cadmium tolerance. *J. Hazard. Mater.* **2014**, *280*, 12–19, doi:10.1016/j.jhazmat.2014.07.048.
84. Bolton, M.D.; Kolmer, J.A.; Xu, W.W.; Garvin, D.F. Lr34-mediated leaf rust resistance in wheat: transcript profiling reveals a high energetic demand supported by transient recruitment of multiple metabolic pathways. *Mol. Plant-Microbe Interact.* **2008**, *21*, 1515–1527, doi:10.1094/MPMI-21-12-1515.
85. Lu, Z.X.; Gaudet, D.; Puchalski, B.; Despins, T.; Frick, M.; Laroche, A. Inducers of resistance reduce common bunt infection in wheat seedlings while differentially regulating defence-gene expression. *Physiol. Mol. Plant Pathol.* **2006**, *67*, 138–148, doi:10.1016/j.pmpp.2005.12.001.
81. Beccari, G.; Covarelli, L.; Nicholson, P. Infection processes and soft wheat response to root rot and crown rot caused by Fusarium culmorum. *Plant Pathol.* **2011**, *60*, 671–684, doi:10.1111/j.1365-3059.2011.02425.x.
86. Desmond, O.J.; Manners, J.M.; Stephens, A.E.; Maclean, D.J.; Schenk, P.M.; Gardiner, D.M.; Munn, A.L.; Kazan, K. The Fusarium mycotoxin deoxynivalenol elicits hydrogen peroxide production, programmed cell death and defence responses in wheat. *Mol. Plant Pathol.* **2008**, *9*, 435–445, doi:10.1111/j.1364-3703.2008.00475.x.
19. Francesconi, S.; Steiner, B.; Buerstmayr, H.; Lemmens, M.; Sulyok, M.; Balestra, G.M. Chitosan hydrochloride decreases Fusarium graminearum growth and virulence and boosts growth, development and systemic acquired resistance in two durum wheat genotypes. *Molecules* **2020**, *25*, 4752, doi:10.3390/molecules25204752.
87. Desmond, O.J.; Edgar, C.I.; Manners, J.M.; Maclean, D.J.; Schenk, P.M.; Kazan, K. Methyl jasmonate induced gene expression in wheat delays symptom development by the crown rot pathogen Fusarium pseudograminearum. *Physiol. Mol. Plant Pathol.* **2006**, *67*, 171–179, doi:10.1016/j.pmpp.2005.12.007.
88. Gaudet, D.A.; Wang, Y.; Frick, M.; Puchalski, B.; Penniket, C.; Ouellet, T.; Robert, L.; Singh, J.; Laroche, A. Low temperature induced defence gene expression in winter wheat in relation to resistance to snow moulds and other wheat diseases. *Plant Sci.* **2011**, *180*, 99–110, doi:10.1016/j.plantsci.2010.07.023.
89. Li, N.; Yin, N.; Niu, Z.; Hui, W.; Song, J.; Huang, C.; Wang, H.; Kong, L.; Feng, D. Isolation and characterization of three TaYUC10 genes from wheat. *Gene* **2014**, *546*, 187–194, doi:10.1016/j.gene.2014.06.020.
90. Xiang, Y.; Song, M.; Wei, Z.; Tong, J.; Zhang, L.; Xiao, L.; Ma, Z.; Wang, Y. A jacalin-related lectin-like gene in wheat is a component of the plant defence system. *J. Exp. Bot.* **2011**, *62*, 5471–5483, doi:10.1093/jxb/err226.
91. Yang, F.; Dong, F.S.; Hu, F.H.; Liu, Y.W.; Chai, J.F.; Zhao, H.; Lv, M.Y.; Zhou, S. Genome-wide identification and expression analysis of the calmodulin-binding transcription activator (CAMTA) gene family in wheat (Triticum aestivum L.). *BMC Genet.* **2020**, *21*, 1–10, doi:10.1186/s12863-020-00916-5.
92. Vicente, R.; Pérez, P.; Martínez-Carrasco, R.; Usadel, B.; Kostadinova, S.; Morcuende, R. Quantitative RT-PCR platform to measure transcriptlLevels of C and N metabolism-related genes in durum wheat: transcript profiles in elevated [CO2] and high temperature at different levels of N supply. *Plant Cell Physiol.* **2015**, *56*, 1556–1573, doi:10.1093/pcp/pcv079.
93. Wang, Z.; Li, W.; Qi, J.; Shi, P.; Yin, Y. Starch accumulation, activities of key enzyme and gene expression in starch synthesis of wheat endosperm with different starch contents. *J. Food Sci. Technol.* **2014**, *51*, 419–429, doi:10.1007/s13197-011-0520-z.
94. Tundo, S.; Janni, M.; Moscetti, I.; Mandalá, G.; Savatin, D.; Blechl, A.; Favaron, F.; D'Ovidio, R. PvPGIP2 accumulation in specific floral tissues but not in the endosperm limits Fusarium graminearum infection in wheat. *Mol. Plant-Microbe Interact.* **2016**, *29*, 815–821, doi:10.1094/MPMI-07-16-0148-R.
78. Tenea, G.N.; Peres Bota, A.; Cordeiro Raposo, F.; Maquet, A. Reference genes for gene expression studies in wheat flag leaves grown under different farming conditions. *BMC Res. Notes* **2011**, *4*, 373, doi:10.1186/1756-0500-4-373.
80. Horevaj, P.; Milus, E.A.; Bluhm, B.H. A real-time qPCR assay to quantify Fusarium graminearum biomass in wheat kernels. *J. Appl. Microbiol.* **2011**, *111*, 396–406, doi:10.1111/j.1365-2672.2011.05049.x.
95. Mudge, A.M.; Dill-Macky, R.; Dong, Y.; Gardiner, D.M.; White, R.G.; Manners, J.M. A role for the mycotoxin deoxynivalenol in stem colonisation during crown rot disease of wheat caused by Fusarium graminearum and Fusarium pseudograminearum. *Physiol. Mol. Plant Pathol.* **2006**, *69*, 73–85, doi:10.1016/j.pmpp.2007.01.003.
22. Francesconi, S.; Mazzaglia, A.; Balestra, G.M. Different inoculation methods affect components of Fusarium head blight resistance in wheat. *Phytopathol. Mediterr.* **2019**, *58*, 679–691, doi:10.13128/Phyto-10942.
